# Machine-Learning Predictions of Critical Temperatures from Chemical Compositions of Superconductors

**DOI:** 10.1021/acs.jcim.4c01137

**Published:** 2024-09-17

**Authors:** Son Gyo Jung, Guwon Jung, Jacqueline M. Cole

**Affiliations:** † Cavendish Laboratory, Department of Physics, University of Cambridge, J. J. Thomson Avenue, Cambridge CB3 0HE, U.K.; ‡ ISIS Neutron and Muon Source, STFC Rutherford Appleton Laboratory, Harwell Science and Innovation Campus, Didcot, Oxfordshire OX11 0QX, U.K.; § Research Complex at Harwell, Rutherford Appleton Laboratory, Harwell Science and Innovation Campus, Didcot, Oxfordshire OX11 0FA, U.K.; ∥ Scientific Computing Department, STFC Rutherford Appleton Laboratory, Harwell Science and Innovation Campus, Didcot, Oxfordshire OX11 0QX, U.K.

## Abstract

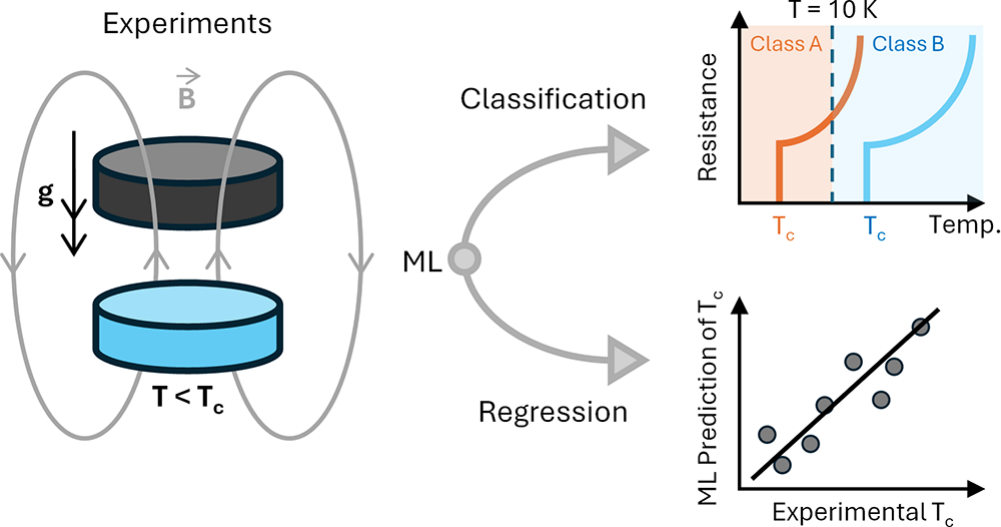

In the quest for advanced superconducting materials, the accurate prediction of critical temperatures (*T*_c_) poses a formidable challenge, largely due to the complex interdependencies between superconducting properties and the chemical and structural characteristics of a given material. To address this challenges, we have developed a machine-learning framework that aims to elucidate these complicated and hitherto poorly understood structure–property and property–property relationships. This study introduces a novel machine-learning-based workflow, termed the Gradient Boosted Feature Selection (GBFS), which has been tailored to predict *T*_c_ for superconductors by employing a distributed gradient-boosting framework. This approach integrates exploratory data analyses, statistical evaluations, and multicollinearity reduction techniques to select highly relevant features from a high-dimensional feature space, derived solely from the chemical composition of materials. Our methodology was rigorously tested on a data set comprising approximately 16,400 chemical compounds with around 12,000 unique chemical compositions. The GBFS workflow enabled the development of a classification model that distinguishes compositions likely to exhibit *T*_c_ values greater than 10 K. This model achieved a weighted average F1-score of 0.912, an AUC-ROC of 0.986, and an average precision score of 0.919. Additionally, the GBFS workflow underpinned a regression model that predicted *T*_c_ values with an *R*^2^ of 0.945, an MAE of 3.54 K, and an RMSE of 6.57 K on a test set obtained via random splitting. Further exploration was conducted through out-of-sample *T*_c_ predictions, particularly those exceeding the liquid nitrogen temperature, and out-of-distribution predictions for (Ca_1–*x*_La_*x*_)FeAs_2_ based on varying lanthanum content. The outcome of our study underscores the significance of systematic feature analysis and selection in enhancing predictive model performance, offering various advantages over models that rely primarily on algorithmic complexity. This research not only advances the field of superconductivity but also sets a precedent for the application of machine learning in materials science.

## 1 Introduction

Superconductivity is a phenomenon in certain materials that is made distinctive by the complete disappearance of electrical resistance and the expulsion of magnetic fields, facilitated by the Meissner effect.^1,2^ Such materials are termed superconductors. Unlike conventional metallic conductors, whose resistance gradually decreases as the temperature approaches absolute zero, superconductors transition abruptly to a superconducting state, enabling the lossless conduction of electricity below a certain critical temperature (*T*_c_). This distinctive behavior underpins the significant potential applications of superconductors across various fields.

However, the existing phenomenological theories of superconductivity are found to be insufficient, primarily due to the considerable theoretical and experimental challenges associated with understanding the intrinsic relationship between superconductivity and both the chemical composition and structural features of these materials. Notably, the mechanisms underlying high-temperature superconductivity in cuprates^3,4^—a class of compounds characterized by crystal planes composed of copper and oxygen atoms—and iron-based families^5−7^ have yet to be fully comprehended. This shortfall highlights a gap in the prevailing theoretical frameworks, necessitating the need for a more thorough understanding to effectively elucidate these complex phenomena.

The exploration of superconductors has traditionally been guided by experimental methods that use trial-and-error approaches. These methods are labor-intensive, operationally costly, and require a sustained level of investment in specialist human capital. Recently, the proliferation of large chemical data sets, propelled by big-data initiatives, has led to a shift toward data-driven materials discovery. The adeptness of data science in processing and analyzing vast, high-dimensional data sets has the potential to streamline the design-to-device pipeline for materials discovery, delivering a level of efficiency unattainable with traditional methods.

Within this data-driven paradigm, the integration of materials informatics with statistical and machine learning (ML) methods is crucial. The typical materials-informatics workflow transforms chemical data into machine-readable formats using feature descriptors, which are subsequently used to train models. These models facilitate the statistical predictions of properties for unseen chemical materials through regression analysis or to categorize materials into specific classes using classification algorithms. The overarching goal is for ML models to learn and predict the intricate relationships between chemical compositions, material structures, and their properties. This approach aims to achieve a level of efficiency and predictive capability that significantly exceeds the constraints of manual analysis.

ML methodologies have already proven highly effective in accurately predicting chemical structures and properties across diverse research domains, including the materials-property prediction of Fermi energy and Poisson ratio,^8^ local magnetic moments,^9^ melting temperature,^10^ among other various properties.^11,12^ Multifidelity modeling strategies that synergize high-throughput computational calculations with experimental measurements have been shown to enhance predictive capabilities, particularly in scenarios where experimental measurements are sparse.^13,14^ ML techniques have proven useful in facilitating automatic material characterization across different spectral analyses.^14,15^

These studies not only showcase the successful application of ML in materials science but also illustrate how ML methods can drive the targeted design of novel materials based on a desired property within a highly complex feature space. By employing predictive modeling to optimize material properties, these techniques increase efficiency and reduce the need for extensive empirical testing. Such approaches can be particularly useful in the study of superconductors with high-*T*_c_ values, where a multitude of complex interactions among ions and their environments pose challenges in predicting superconducting properties.

Early foundational research in this field was pioneered by Villars and Phillips^16^ and Rabe et al.,^17^ who successfully clustered 60 superconductors with *T*_c_ exceeding 10 K into three distinct groups. The classification system developed by Villars et al. organized crystal structures, ranging from binary to quaternary compounds, highlighting their tendencies to form specific types of alloys. This framework was leveraged to analyze specific groups of compounds such as quasicrystals, high-*T*_c_ superconductors, and ferroelectrics. The results helped to identify trends and develop an automated approach to predict new materials.

Building upon this groundwork, Isayev et al.^18^ revisited this clustering analysis by raising the threshold *T*_c_ to 20 K. They employed a random-forest algorithm combined with simplex fragments to examine structural and electronic properties, which led to the identification of optimal clustering descriptors. This analysis culminated in the development of systematic strategies that predict new materials based on their crystallographic and electronic characteristics. Other concurrent studies have focused on particular materials^19,20^ and specific families of superconductors,^21,22^ further delineating the complex relationships that underpin superconductivity across various material systems.^23^

Additional studies include: (i) the use of statistical methods to explore potential correlations between *T*_c_ values of metallic elements and their normal state properties, specifically for elements within the first six rows of the periodic table;^24^ (ii) the use of a sequential learning framework on superconductors to evaluate the efficacy of random guessing or trial-and-error approaches in materials research.^25^ This analysis has not only highlighted the inherent limitations of traditional methodologies but also emphasized the transformative potential of ML in advancing research and fostering innovation in the field of superconductivity.

In contrast to the aforementioned studies, which only included several hundred compounds, Stanev et al.^26^ analyzed approximately 16,000 chemical compositions to study superconductors. They developed both classification and regression models using variants of the random forest algorithm^27^ to categorize materials that are likely to exhibit *T*_c_ values greater than 10 K and to predict *T*_c_ values based on the chemical composition of the materials, respectively. The data for this study were sourced from the SuperCon database, maintained by the Japanese National Institute for Materials Science, which provides a comprehensive collection of superconductors. In their classification analysis, Stanev et al. achieved an accuracy and F1 score of about 0.92. In the regression analysis, they reported a coefficient of determination (*R*^2^) of 0.885 in the *T*_c_ space and 0.88 in the ln(*T*_c_) space on their out-of-sample evaluation. Both analyses involved a train-to-test split ratio of 85%:15%.

In the realm of deep learning, the SuperCon data set was used by Konno et al.^28^ to apply a deep convolutional neural network that analyzes the periodic table. This approach enabled the identification of patterns among chemical elements to predict *T*_c_. In their regression task, they achieved an *R*^2^ of 0.92 on the test set, using a train-to-test split ratio of 95%:5%. For the classification task, which aimed at identifying materials with a *T*_c_ value above 10 K, they integrated an additional data set^29^ to assess the model’s performance. They reported an accuracy of 0.95, an F1 score of 77%, and an area-under-the-curve of the receiver-operating-characteristic curve (AUC-ROC) of 0.94, employing a temporal separation testing scheme. Additionally, Pereti et al.^30^ engaged the SuperCon data set to conduct both supervised classification and regression of superconductors using DeepSet models.^31^ They initially reported an *R*^2^ of 0.92 and a root-mean-square error (RMSE) of 9.5 K on their test set. They improved the model performance to an *R*^2^ of 0.93 and an RMSE of 9 K by implementing an ensemble technique with a train-to-test split ratio of 80%:20%. In their classification task, which aimed to discriminate superconductors with a threshold of 10 K, they achieved an accuracy of 0.84.

This study describes a new approach to predict the *T*_c_ of superconductors using only their chemical compositions. The objective is to deliver more accurate and transparent data-driven designs of superconductors through a systematic approach for feature engineering, analyses, selection, and model optimization.^32^ Our proposed Gradient Boosting Feature Selection (GBFS) framework employs a distributed gradient-boosting algorithm, enriched with comprehensive data and statistical analyses. This framework also incorporates multicollinearity reduction techniques to identify a subset of features that are salient to the target variable or class within a highly complex feature space, thereby minimizing feature redundancy and maximizing their relevance. The proposed methodology aims to provide a robust framework for effectively discerning the properties of superconductors, leveraging advanced analytical techniques to streamline the design process.

Our workflow demonstrates broad applicability across different domains of materials science, using data from density functional theory (DFT), experimental measurements, or a combination of both to accurately predict material-property relationships.^13,14,32−34^ Here, we extend the application of the GBFS framework to include training a classifier to distinguish materials with a *T*_c_ value greater than 10 K. We also trained four separate regression models to predict the *T*_c_ values of superconducting materials, employing a training set derived from literature-based experimental data. The initial model is applied universally across the data set, encompassing all classes of superconductors. To deepen our understanding of the specific behaviors of different superconducting families, we subsequently constructed additional regression models, each tailored to a distinct family of superconductors. These ML models reveal the unique superconducting characteristic of each class, providing insights into their diverse superconducting properties. The GBFS framework not only facilitates the prediction of superconducting temperatures but also enhances our understanding of the interactions among features during the prediction process. This improves our comprehension of the factors that either promote or impede superconductivity.

Furthermore, we benchmark the performance of our models against state-of-the-art ML methods that also predict *T*_c_ values. To ensure a rigorous comparison with state-of-the-art approaches and to adhere to a stringent validation criteria, we confined our feature selection strictly to descriptors that are derived from the chemical compositions of materials. This decision also recognizes the frequent absence of detailed crystallographic data in experimental reports of *T*_c_, while chemical composition information remains readily accessible.

Overall, we will demonstrate the effectiveness of our GBFS approach within this research domain, while emphasizing the advantages of employing a systematic approach to feature analysis and optimization over a reliance on complex modeling techniques. We will illustrate how common off-the-shelf algorithms, when trained on features selected and engineered through the GBFS framework, can surpass the performance of models reported in the existing literature. Moreover, our approach provides valuable insights into the interactions among features and their relevance to the target variable or class, thereby enhancing the predictive accuracy and interpretability of our models. This reinforces the significance of meticulous feature analysis and selection in advancing the field of materials informatics. To aid further research in this field, we have made available the code utilized in this study, alongside the database of *T*_c_ values and their chemical compositions. They are accessible via https://github.com/Songyosk/SuperconductivityML.

## 2 Methods

### 2.1 Data Sources

We employed the Supercon data set that contains approximately 16,000 published experimental superconductivity records. The data set was initially compiled by Stanev et al.^26^ from data curated by the Japanese National Institute for Materials Science. The data set lacks structural information and includes around 4000 entries with either no reported superconducting transition temperature or a reported lack of superconductivity (*T*_c_ = 0 K). Despite these limitations, the data set remains a valuable resource as it includes a diverse array of superconductors. There are also *T*_c_ records of superconductors with minor stoichiometric variations, which provide insight into the effects of doping—a critical factor in the optimization of *T*_c_. For training and evaluating our ML models, we maintained a train-to-test split of 85%:15%, aligning with the methodology of Stanev et al.^26^ This data set is one of the most comprehensive compilations of superconductivity data that is publicly available, offering detailed *T*_c_ records across various families of superconducting materials.

### 2.2 Feature Descriptors

We constructed a high-dimensional feature vector for each chemical material by employing composition-based descriptors. This step leveraged the featurization capabilities of Matminer^35^ and Pymatgen.^36^ These feature vectors served as the input for our GBFS workflow. We augmented these descriptors with statistical measures that were derived from unique elemental properties of each chemical entity. The calculation of these statistical features was supported by data from multiple sources, including Magpie^37^ and Pymatgen,^36^ and enriched with predictive insights from the Deml^38^ database and the MatErials Graph Network^39^ (MEGNet) model. We used the neural-network embeddings from the MEGNet model to enhance elemental characterization, enabling transfer learning from previously trained models.

### 2.3 GBFS Workflow

**Figure 1:**
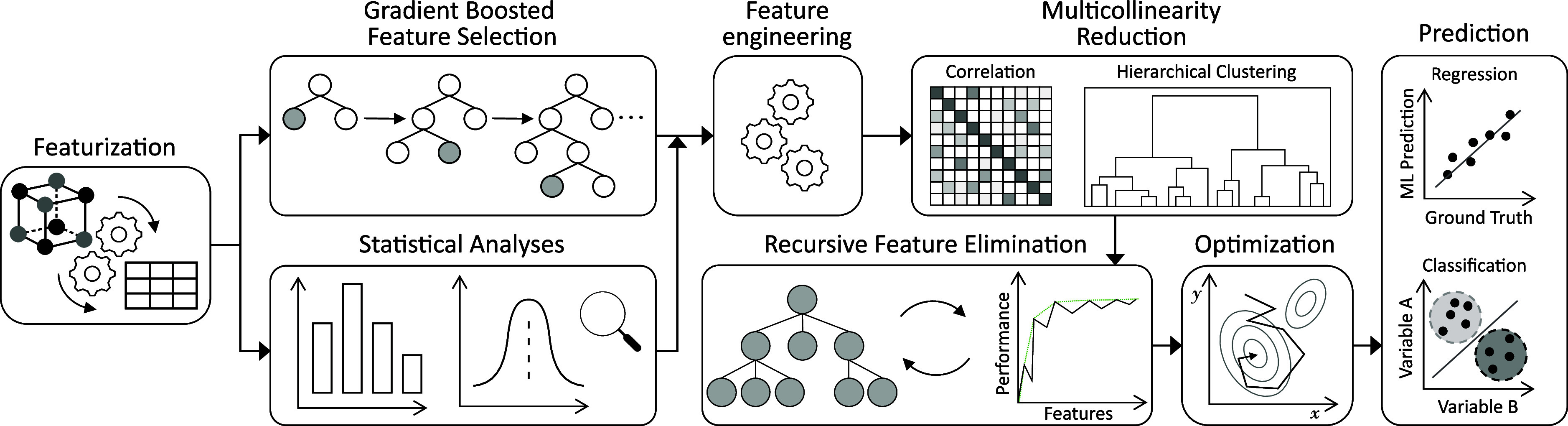
Schematic diagram of the operational workflow employed in this study. For a comprehensive description, please refer to the cited source.^32^ Part of the figure is reproduced with permission from ref (32).

Figure 1 depicts our overarching GBFS workflow. The GBFS workflow used in this study is comprised of several steps that are designed to optimize model performance, focusing on the feature level in a systematic manner. Our workflow incorporates: (i) a gradient-boosting framework to select a subset of features that is most relevant to the target variable or class, (ii) statistical analyses to discern those that are statistically significant to the target variable or class, (iii) a feature-engineering step to create supplemental features, (iv) a two-step multicollinearity reduction step that is aimed at reducing feature redundancy using correlation and hierarchical-clustering analyses, (v) recursive-feature elimination, a greedy optimization algorithm, for further feature refinement, and (vi) Bayesian optimization to perform hyperparameter optimization of ML models.

While Jung et al.^32^ provide a comprehensive description of each component within our GBFS workflow, we herewith present a summary of its key attributes to ensure clarity and highlight how our approach diverges from previous studies. Our modeling approach is notably systematic and minimizes human intervention during both the feature selection and model development phases. The process begins with the compilation of an extensive list of exploratory features. We then assess the contribution of each feature by calculating the associated loss reduction or variance gain caused by each feature; the result of which provides a preliminary feature ranking that is based on the derivatives (i.e., the gradients) of a loss function. In parallel, we perform a series of statistical tests and analyses that are rooted in probability and information theory. These two independent stages work in tandem to identify the most relevant features for predicting the target variable—in this case, the *T*_c_ values. Once these key features are identified, they serve as the foundation for generating additional features. Our default method for this stage is a brute-force approach that does not require domain-specific knowledge, although manual intervention can be introduced at this point to guide the feature-engineering process. The most salient and statistically significant features, along with the newly engineered features, undergo evaluation for multicollinearity to ensure robust model performance.

To mitigate multicollinearity, the process begins by eliminating highly correlated features using a predefined threshold applied to a correlation matrix. Hierarchical clustering analysis is then used to group similar features together. A linkage threshold is established, enabling the algorithm to automatically select a representative feature from each cluster. This approach is based on the premise that one representative feature can capture the same information as the others in the cluster, thereby reducing the complexity of the feature space while preserving essential information. Subsequently, recursive-feature elimination is performed using a greedy-based search algorithm that methodically removes features until either a specified number of features is retained or no further decline in model performance is detected. Concurrently, permutation-importance analysis is conducted, which involves randomly shuffling the values of a single feature to assess its impact on a set of performance metrics. This step is critical for understanding the influence of individual features on the predictive capability of the model.

This comprehensive process culminates in a systematic selection of a refined subset of features, which are then used to conduct Bayesian optimization. During the hyperparameter-optimization stage, the optimal model architecture is autonomously identified using only the training set. This approach allows the optimization algorithm to systematically select the optimal values for the hyperparameters, creating a seamless end-to-end process that operates without the need for any human intervention, thereby eliminating potential human bias. Once the final predictive model is optimized, it is evaluated using the test set—this being the first and only instance where the test data are utilized. This methodology ensures that our ML model is both robust and effective, trained within a feature space that has been meticulously curated by the algorithms, thereby eliminating human bias. The pseudocode for Bayesian optimization is provided in Supporting Information 1.

The strategic selection of features optimizes their contribution to the predictive accuracy of the model, while minimizing the effects of high correlations and redundancy among the input features. By simplifying the feature space, our modeling approach mitigates potential overfitting and inherently incorporates regularization to enhance model generalization. This underscores the sophisticated and reliable nature of our systematic analytical process. For a comprehensive understanding of this methodology, readers are referred to its detailed description provided by Jung et al.^32^

In our implementation, materials-property predictions were made solely based on the chemical composition of materials. Our approach strategically trains the model to remain agnostic to crystalline polymorphs, thereby eliminating the need for 3-D crystallographic data. Such an approach enables a systematic analysis across a diverse array of superconducting materials. The decision was driven by the frequent absence of comprehensive crystallographic information in the scientific literature, in contrast to the ready availability of chemical compositions. Furthermore, the inclusion of 3-D crystallographic data would not only deplete the data set significantly but also necessitate such information for future predictions, thus severely constraining the utility of the predictive model and introducing a bottleneck in the materials prediction pipeline. Nevertheless, it is important to acknowledge that relying exclusively on the chemical composition of materials has its limitations. Specifically, the model cannot differentiate between materials which have multiple stable phases, each with distinct physical properties.

### 2.4 Evaluation Metrics

The performance of the classification model that was trained through our GBFS workflow is assessed using the area-under-the-curve of the receiver-operating-characteristic curve (AUC-ROC), overall accuracy, and the F1-score. The F1-score, calculated as the harmonic mean of precision and recall, provides a balance between these two metrics, as detailed by the following formulas:
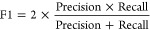
1
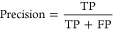
2
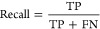
3
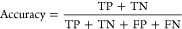
4where TP and TN are true positive and true negative, and FP and FN are false positive and false negative, respectively.

We evaluated the performance of our regression analysis using several metrics, including the mean-absolute error (MAE), the mean-squared error (MSE) and Hamming loss (HL), which quantifies the fraction of labels that are incorrectly predicted. Additionally, we assessed the model’s explanatory ability using the coefficient of determination (*R*^2^), which quantifies the proportion of variance in the dependent variable that is predictable from the independent variables and can be computed as the square of the Pearson correlation coefficient (*R*):
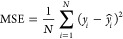
5
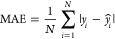
6
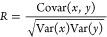
7where *y*_*i*_ represents the observed values, *ŷ*_*i*_ denotes the predicted values from the model, and *N* is the total number of observations. The covariance is given by
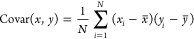
8with  and  representing the variance of the independent variable *x* and dependent variable *y*, where  and  are the mean values of *x* and *y*, respectively. The coefficient *R* ranges between −1 and 1, indicating the strength and direction of a linear relationship between the variables. In alignment with established practices in the literature, we used these metrics to ensure consistency in our evaluation across different regression and classification tasks.

## 3 Results and Discussion

### 3.1 Classification of Superconductors

#### 3.1.1 Overall Performance Evaluation

In our classification analysis, we excluded chemical compositions that lacked *T*_c_ data. Such compositions were considered inappropriate for analysis under the assumption of *T*_c_ = 0, as this potentially imposes an inadequate presumption for these compounds. For chemical entries that were documented multiple times, we calculated the median *T*_c_ value. The median was selected over the mean due to its relative robustness against outliers. Our data set comprised approximately 12,000 unique chemical compositions.

We assigned binary classification labels based on a critical temperature threshold of 10 K. (i.e., *T*_thres_ = 10 K). This threshold was chosen as it effectively reduces class imbalance and optimizes classification metrics (refer to eqs 5–7). This approach aligns with the findings of Stanev et al.^26^ and is supported by earlier studies,^16,17^ thus ensuring an equitable distribution of classification labels. To further mitigate the effects of class imbalance at both the model development and evaluation phases, we applied the smoothed random oversampling (smoothed-ROS) technique with a shrinkage factor of 0.35. Smoothed-ROS is an extension of random oversampling with Gaussian noise. The addition of stochastic noise helps to prevent model overfitting on specific feature values by increasing the intraclass sample variability, thereby improving model generalization. Our results indicated that smoothed-ROS alleviates potential learning biases most effectively when compared to other oversampling strategies.

For the partitioning of our data set, we implemented a stratified train-to-test split to preserve the class ratio consistency within the training and test sets as observed in the original data set. Each chemical composition was randomly selected from the initial data pool during this process. An 85%:15% ratio was maintained between the training and test sets. The resulting training set was subjected to feature processing through the GBFS workflow, which was instrumental in optimizing our model’s performance.

**Figure 2:**
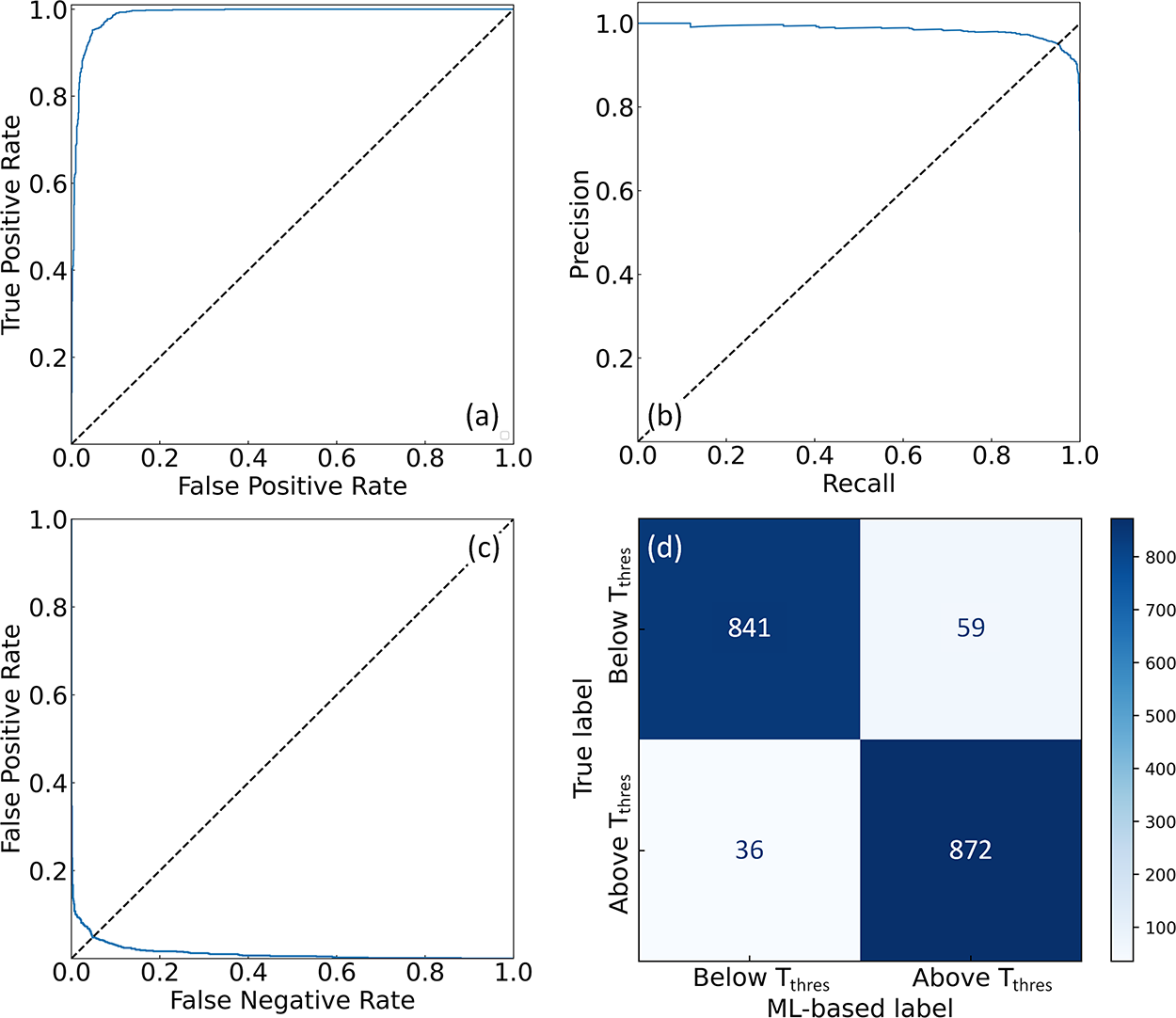
Classification performance on the test set. (a) Receiver operating characteristic (ROC), (b) precision-recall (PR), (c) detection error trade-off (DET) curves and (d) confusion matrix. An AUC-ROC of 0.986, an average precision (AP) score of 0.919 and an equal error rate (ERR) of 0.05 are realized.

The classification model we developed via the GBFS workflow demonstrated excellent efficacy, outperforming several benchmarks in the literature, as summarized in Section 1. Starting with an initial set of approximately 730 exploratory features and about 90 engineered features, the model identified a subset of the 29 most pertinent features. It achieved a weighted average F1-score of 0.912, an AUC-ROC of 0.986, an average precision (AP) of 0.919, and an accuracy of 0.947. The performance of the classification model is further illustrated in Figure 2a–c, along with the confusion matrix in Figure 2d. A detailed breakdown of precision, recall, and F1-score metrics for each target label is presented in Table 1. Here, we use the AP score to summarize the precision-recall (PR) curve shown in Figure 2b, rather than the area under the curve. This decision was based on the potential overestimation of the area under the PR curve when calculated using the trapezoidal rule via linear interpolation. The AP is calculated as the weighted average of precision at each classification threshold, with the weights being the increments in recall from the previous threshold. These measures indicate that our classification model is highly discriminative, effectively distinguishing chemical compositions that are likely to exhibit *T*_c_ above 10 K.

**Table 1:** Summary of the Performance Metrics for the Classification of Superconductors by Their *T*_c_ Values Relative to Threshold *T*_thres_ = 10 K

	precision	recall	F1-score
below *T*_thres_	0.959	0.934	0.947
above *T*_thres_	0.937	0.960	0.948
macro average	0.948	0.947	0.910
weighted average	0.948	0.947	0.912

These results were realized without adopting specific modifications or treatments to account for polymorphism—the presence of distinct 3-D crystal structures of the same chemical compound. Our approach strictly trains the model to remain indifferent to polymorphic variations. The ML model exhibits a high level of predictability, notwithstanding its ignorance of the various 3-D crystal structures that a compound may manifest, owing to the myriad ways in which chemical elements can arrange within unit cells of the crystalline lattice of each material.

We now explore specific evaluation metrics in Table 1. The table summarizes the performance metrics for our classification model that is designed to predict whether observations fall above or below *T*_thres_ = 10 K. The metrics displayed include precision, recall, and F1-score for the categories ‘Below *T*_thres_’ and ‘Above *T*_thres_’, as well as aggregated statistics in the form of macro and weighted averages across these categories.

Precision measures the proportion of true positives relative to all positive predictions made by the model. A precision of 0.937 for instances classified as ‘Above *T*_thres_’ suggests that 93.7% of the predictions made by the model for being above the threshold were indeed correct, with a minimal incidence of false positives. Conversely, recall assesses the percentage of actual positive instances correctly identified by the model. A recall of 0.960 for the ‘Above *T*_thres_’ category indicates that the ML model successfully identified 96.0% of true positives (i.e., superconductors with *T*_c_ > 10 K). For predictions categorized as ‘Below *T*_thres_’, the model demonstrates a precision of 0.959, affirming its high accuracy in predicting true negatives. The corresponding recall of 0.934 signifies that the model correctly identified 93.4% of all actual negatives. These metrics underscore the model’s capacity for distinguishing between the two target classes, ensuring reliable performance in applications where precise threshold-based classification is vital.

The F1-score, calculated as the harmonic mean of precision and recall (as delineated in eq 5), serves as a comprehensive metric that encapsulates the balance between these two evaluation measures. An F1-score nearing 1 denotes an optimal equilibrium between precision and recall. Notably, our ML model afforded F1-scores that are commendably high in both categories, with 0.947 for ‘Below *T*_thres_’ and 0.948 for ‘Above *T*_thres_’, suggesting that the model effectively balances the trade-off between precision and recall. The results attest to the highly discriminative ability of our classifier, particularly highlighted by the substantial recall rate of 0.960 for the ‘Above *T*_thres_’ category. This score is significant as it emphasizes the model’s proficiency in accurately identifying the majority of positive cases, which is often critical in applications where there are severe consequences of failing to detect positive instances.

Table 1 also presents the macro and weighted averages for precision, recall, and F1-score. The macro average calculates the mean performance across both categories, irrespective of class size. Conversely, the weighted average incorporates the support of each class, accounting for the number of instances in each category, which aligns performance evaluation with class prevalence. These averages reflect good model performance across both categories, ensuring a balanced model output regardless of class distribution. The close correspondence between the macro and weighted averages suggests that the class distribution does not adversely affect the model’s performance metrics. This consistency is indicative of excellent model generalization across different levels of class imbalance. Moreover, the high level of uniformity across metrics is likely due to the strategic adjustment of the *T*_thres_ value and the application of an oversampling technique in the training set. Specifically, the use of the smoothed-ROS method ensured equal representation of each class by generating synthetic feature vectors that mimic the characteristics of minority classes within the high-dimensional feature space. Lastly, a 10-fold cross-validation process afforded an F1-score of 0.95 ± 0.01. These performance metrics confirm the robustness and reliability of our classifier in threshold-based decision-making scenarios.

#### 3.1.2 Gradient Boosted and Statistical Feature Selection Workflow

We now turn our attention to the results that stem from each component of the GBFS workflow, as depicted in Figure 1. Employing the GBFS methodology, a refined subset of 29 features was selected from an initial set of 730 exploratory features and an additional 90 engineered features. The subsequent discussion offers a comprehensive analysis of the results achieved through our feature-selection process.

##### 3.1.2.1 GBFS Process

**Figure 3:**
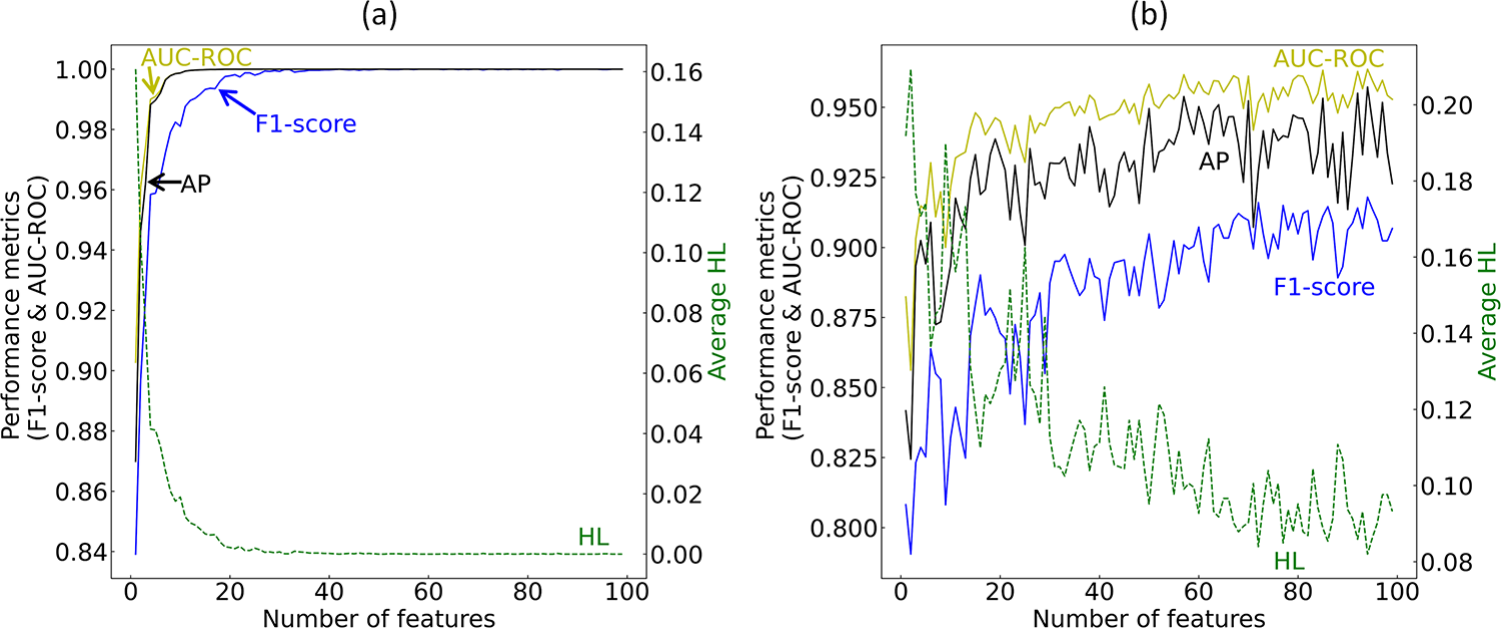
Result of gradient-boosting feature selection for the classification of superconductors by their *T*_c_ values relative to *T*_thres_ = 10 K, detailing the performance of GBDTs on (a) the training set and (b) the validation set. Classification model was trained recursively, starting with the most relevant feature that was based on the realized total loss reduction. This approach involved progressively incorporating an increasing subset of features to evaluate the impact on model performance on both training and validation sets.

The training of gradient-boosted decision trees (GBDTs) involved a recursive process that incorporated progressively larger subsets of features, guided by the convergence of key performance indicators—AUC-ROC, AP, and F1-score. The significance of each feature was determined based on the total loss reduction that was achieved during the ML training process. The effectiveness of the classification model during this feature-selection phase, as evaluated on both the training and validation sets, is depicted in Figure 3.

The performance metrics over the training set stabilized after including approximately 30 features. Conversely, these metrics required a considerably larger set of features to reach convergence over the validation set, necessitating around 70 features. This discrepancy aligns with expectations, as performance commonly declines and exhibits increased fluctuations on an out-of-sample validation set. The variation is partly attributed to the smaller size of the validation set, which was randomly selected from the larger training set. At this stage, our GBFS workflow did not specifically address potential multicollinearity among the exploratory features. The presence of multicollinearity can result in a more uniform distribution of total loss reduction across correlated features, thereby masking the true impact of individual features on the target variable. Based on these observations, we anticipated that a feature set comprising somewhere between the first 30 and 70 features, selected according to total loss reduction, would be necessary to train a classifier that can effectively discriminate between the target classes. The results indicate that statistical measures associated with properties such as periodic table column number, molar volume, thermal conductivity, melting point or temperature, and electronegativity among the constituent elements of a chemical compound accounted for some of the most substantial reductions in loss during the initial training of GBDTs.

##### 3.1.2.2 Feature Analyzes and Feature Engineering

Concurrent with the recursive training of GBDTs, we conducted a comprehensive suite of feature analyses, employing methodologies distinct from those used in our regression study. For the classification task, we leveraged an array of statistical tests and methods, including a generalization of the one-way analysis of variance (ANOVA) F-test, Pearson’s chi-squared test, mutual information (MI) analysis, and discriminant analysis via logistic regression. Detailed descriptions of these methodologies and their applications are provided by Jung et al.^32^

Statistically significant features identified include statistical measures, such as the average deviation and the range of properties of: electronegativity, melting points, molar volume, atomic mass, Goldschmidt’s volume per atom, and the number of valence electrons among the constituent elements of a chemical compound. This selected set of attributes, derived from elemental properties and electronic characteristics, has demonstrated effectiveness in statistically differentiating between the target classes. These features highlight the role that variations in fundamental chemical and physical properties play in influencing the behavior and functionality of materials, thus providing a firm foundation for predictive modeling in materials science.

The selected features were incorporated into an existing list of attributes that had previously contributed to significant loss reduction in the earlier selection stage. To expand this set further, a brute-force method was used to derive an additional 90 features, resulting in a comprehensive preliminary data set comprising 140 features. These were prepared for subsequent downstream analyses, which precedes the final training and optimization of the predictive model. Our approach not only enriches our feature set but also enhances the potential predictive power of our classification model, independent of prior domain-specific knowledge. This strategy ensures that the model development is both data-driven and fully systematic, requiring no human intervention during the selection process.

It is worth noting that the concept of MI was employed to quantify the degree of mutual dependence between two features, specifically measuring the amount of information, or entropy, gained about one feature through the observation of another. MI evaluates the deviation between the joint distribution of a pair of features and the product of their individual marginal distributions. A higher MI value suggests a stronger mutual dependency between the two variables. In our study, an MI estimator based on entropy estimations from K-nearest neighbor distances was employed. The findings indicated that the highest entropy gain was associated with the range of melting points among the constituent chemical elements, as sourced from Pymatgen.^36^ Nevertheless, it is important to highlight that estimating these distributions becomes particularly challenging in high-dimensional spaces, especially when the sample size is small relative to the number of dimensions. Such a constraint can lead to substantial variability in probability estimates, consequently affecting the reliability of the estimated information gain in MI analysis.

##### 3.1.2.3 Multicollinearity Reduction, Permutation Analysis, and Recursive Feature Elimination

In the next phase of our GBFS workflow, we implemented a multicollinearity reduction strategy within the data set. This followed assessing the permutation importance of the selected features and conducting recursive-feature elimination to determine the final subset of features. These steps are crucial for the Bayesian optimization of the final ML model, ensuring that only the most relevant and nonredundant features contributed to model development.

**Figure 4:**
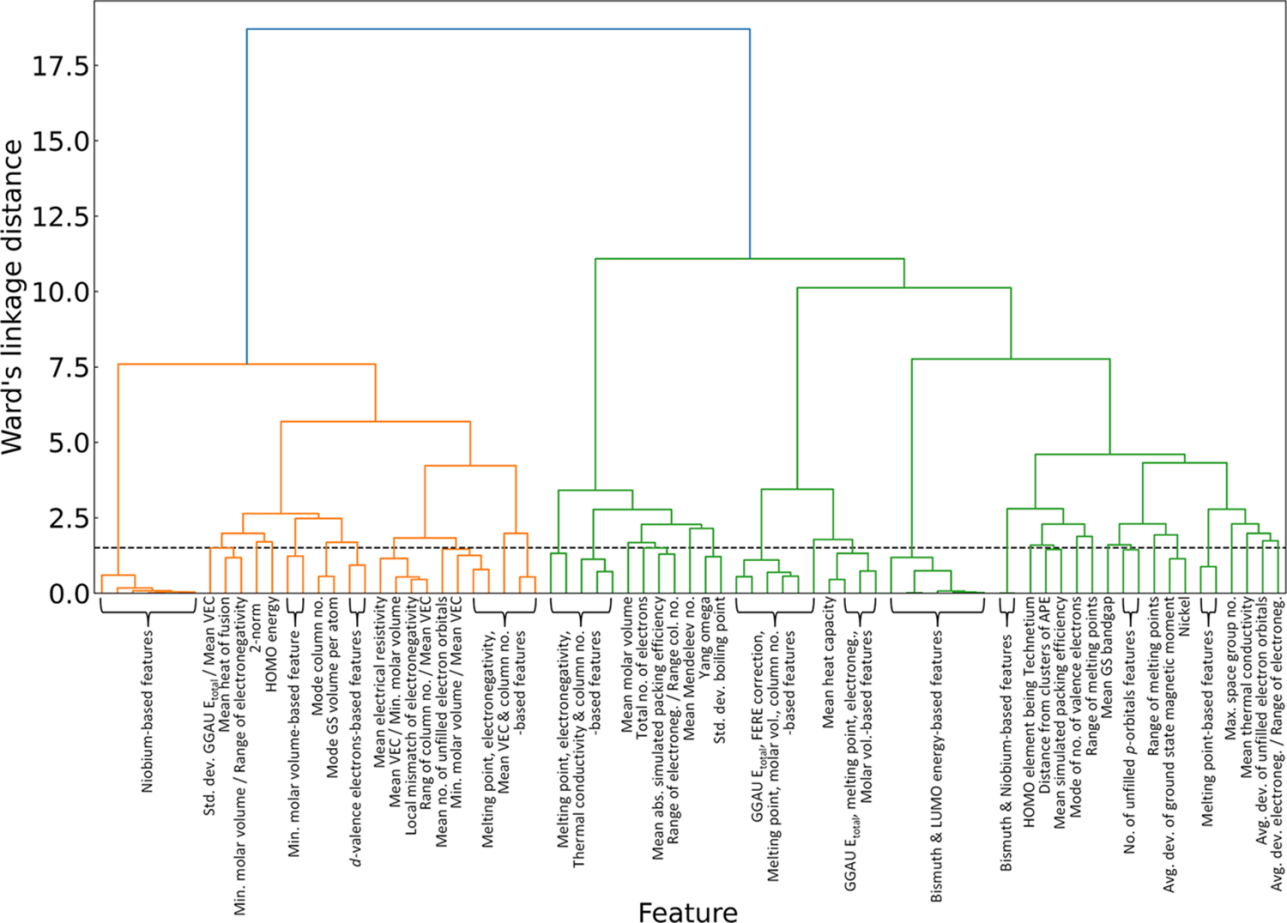
Dendrogram illustrating the results of hierarchical-agglomerative clustering, conducted on the remaining 77 features after applying the correlation analysis. Dashed horizontal line in the dendrogram signifies the established distance threshold of 1.5 units according to Ward’s linkage criterion.

To address the effects of multicollinearity within the data set, features exhibiting a correlation coefficient of 0.8 or higher were systematically excluded. This step reduced the feature set to 77 distinct attributes. Further remediation involved the use of hierarchical-cluster analysis. We used the Spearman rank-order correlation coupled with Ward’s linkage method, setting a distance threshold of 1.5 units. The hierarchical-agglomerative clustering resulted in the retention of 34 features, with only one feature selected from each cluster to minimize redundancy. The optimal-distance threshold was identified using the Elbow method. The result of this clustering is illustrated in the dendrogram depicted in Figure 4, which illustrates the formation of clusters as one progresses upward along the dendrogram.

**Figure 5:**
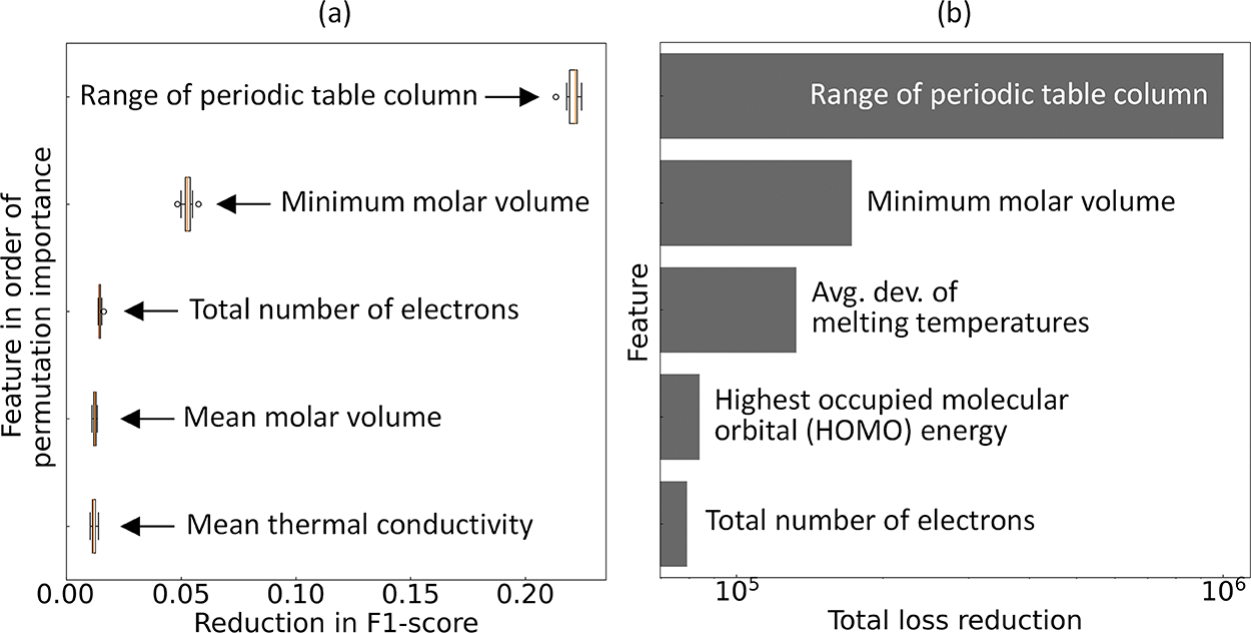
(a) Permutation-based feature importance and (b) feature relevance plots for the classification analysis of superconductors categorized by whether their *T*_c_ values fall below or above *T*_thres_. Figure presents the plots illustrating the five most significant features.

The outcome of the 10-fold permutation feature-importance analysis is depicted in Figure 5a. Permutation-feature importance measures the impact on model performance when the association between a single feature and the target is disrupted by randomly shuffling the feature value. The resulting deterioration of model performance thus reflects the model’s dependency on the specific feature. According to the 10-fold feature permutation analysis, the most relevant feature is the range of periodic table column numbers among the constituent elements in a chemical composition. This is followed in importance by the minimum molar volume, the total number of electrons, the mean molar volume, and the mean thermal conductivity of the constituent elements. We observed a clear consistency of these findings with those realized via the independent statistical analyses, where three of these attributes were previously identified.

Following the reduction of multicollinearity, the optimal subset of features was ascertained through a 10-fold recursive-feature elimination process, which employed the weighted F1-score as the metric for performance assessment. Recursive-feature elimination is a greedy optimization technique designed to identify the most effective subset of features based on a chosen metric. This process entails constructing models recursively and identifying the most pertinent feature at each iteration. This modeling approach continues with the remaining features until all have been evaluated. Features that do not enhance the performance of the chosen metric are systematically eliminated. Such a process led to the selection of the final subset of 29 features. We emphasize that the selected features are chosen from an initial pool of ca. 730 original features and 90 engineered features via our GBFS workflow. These 29 features bear the highest relevance to the target classes without any prior knowledge of the research domain.

It is crucial to note that the aforementioned element embeddings in Section 2.2, derived from graph-neural-network models such as MEGNet, effectively capture the chemical periodicity and inherent trends of the periodic table. Although interpreting these individual embeddings can be challenging, their value has been proven in scenarios involving transfer learning. Specifically, embeddings generated from a model trained on an extensive data set can be used to enhance the predictive accuracy of other models that are trained on more constrained data sets. In our study, we employed these pretrained embeddings to improve the classification accuracy, achieving performance metrics: a weighted average F1-score of 0.954, an AUC-ROC of 0.988, an AP of 0.928, and an overall accuracy of 0.954. These outcomes are comparable to those achieved without the incorporation of these embeddings. Notably, two of the top five most relevant features were substituted with these embeddings, demonstrating their utility in transfer-learning settings. Henceforth, our classification analysis will concentrate on the results obtained without the use of these embeddings to ensure greater interpretability of the feature interactions in our final prediction.

##### 3.1.2.4 Model Optimization and SHAP Analysis

A two-phase optimization strategy was adopted to determine the hyperparameters of the final classifier. Initially, the model’s hyperparameters were tuned using the grid-search method, allowing for an extensive exploration of a broad hyperparameter space. This would otherwise be computationally intensive to navigate. The preliminary stage efficiently identified the specific region of the hyperparameter space that was suitable for subsequent Bayesian optimization. Bayesian optimization is particularly advantageous for optimizing objective functions that lack closed-form expressions, are expensive to evaluate, and produce noisy outcomes during evaluation. Such a method enhances efficiency in identifying optimal settings by strategically navigating the hyperparameter space by balancing the trade-off between exploration and exploitation via an acquisition function.

Figure 5b illustrates the five features that contributed the most to the total loss reduction in the final ML model (i.e., the feature-relevance ranking), as per the target class predictions. These features are largely consistent with those identified in the feature permutation-importance analysis, with a notable distinction being the inclusion of the average deviation of melting temperatures among constituent elements in the chemical composition, and the HOMO energy. The result highlights the dynamic nature of feature relevance across different stages of model development and optimization.

**Figure 6:**
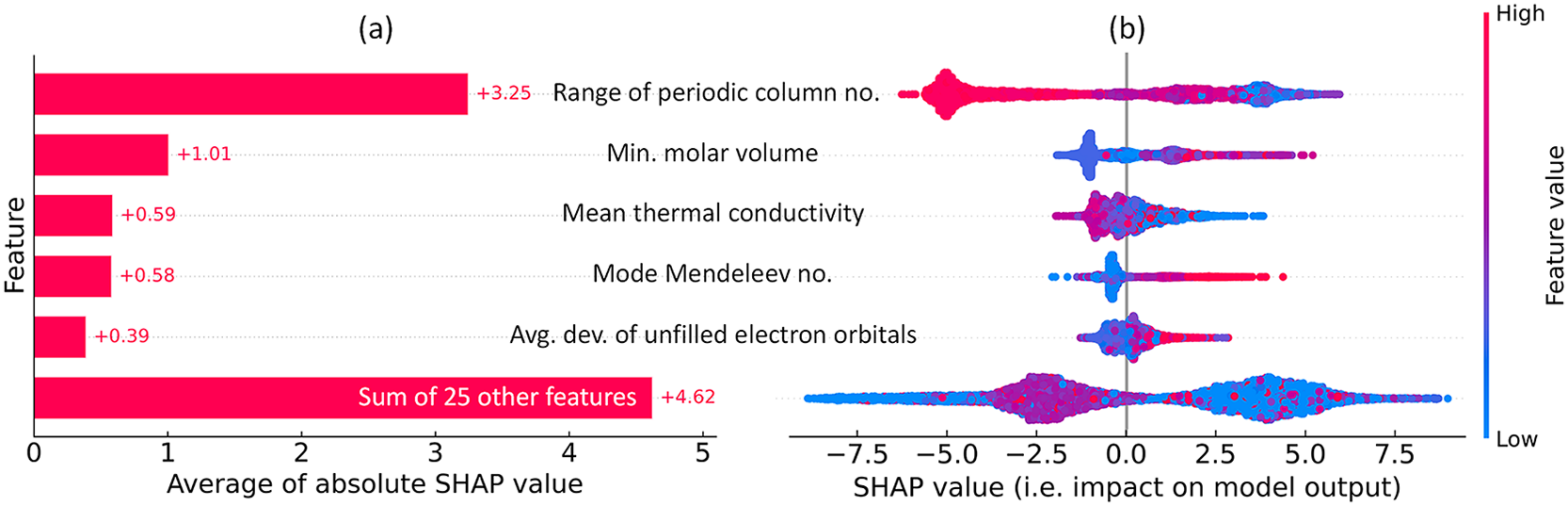
Result generated using the SHapley Additive exPlanations (SHAP) framework. (a) Average contribution plot, represented by the mean absolute SHAP value, of the five features identified as having the most significant impact on the model’s output. Positive SHAP value indicates a positive contribution toward the ‘Below *T*_thres_’ class in the classification analysis. (b) Beeswarm plot visualizes the effect of these features on the model’s output by displaying each data instance as a single point alongside its SHAP value on the *x*-axis, while alignment on the *y*-axis corresponds to the features in (a). Color scheme of each point reflects the original feature value, and the broadening shows the density of instances.

An independent analysis of feature contributions was carried out using the SHapley Additive exPlanations (SHAP) framework,^40^ which is a game-theoretical method designed to explain the output of ML models. Figure 6a presents the average contribution plot, which quantifies the mean absolute SHAP values of the top five features that significantly influence the model’s output. Furthermore, the accompanying beeswarm plot in Figure 6b visualizes the impact of these features on model performance by displaying each instance as an individual data point along with its corresponding SHAP value on the *x*-axis. These results align well with those generated through our GBFS workflow (Figure 5b), further validating the efficacy of our modeling strategy. The findings also imply that the statistical measures of periodic table column number, molar volume, thermal conductivity, and unfilled electron orbitals among the constituent elements play pivotal roles in training a ML model that is capable of effectively discriminating between target classes; that is to categorize superconductors based on a predefined *T*_c_ threshold.

**Figure 7:**
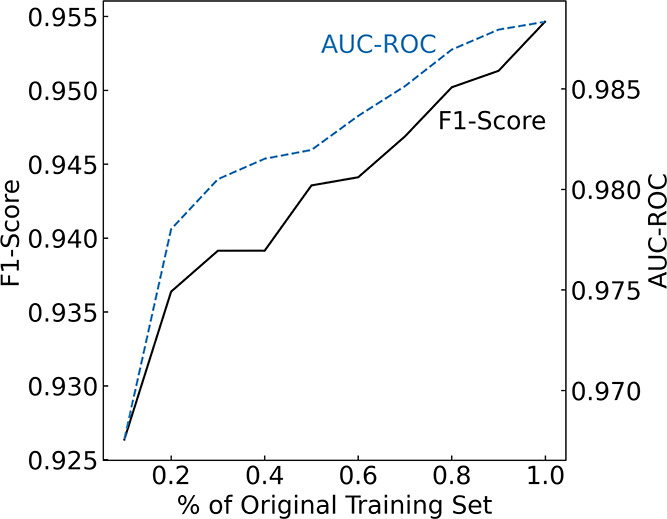
Evaluation of F1-Score and AUC-ROC conducted by training our ML model on increasingly larger subsets of the original training set, where the data were randomly sampled.

We recognize the importance of investigating how variations in data set size affect model performance. This evaluation is vital for determining if the data set size is a constraining factor, or if there is an optimal size of the training set. Our analysis calculated the F1-score and AUC-ROC values for increasingly larger subsets of the original training set, which were selected through random sampling. As depicted in Figure 7, there is a consistent improvement in performance as the size of the training set increases, though these metrics do not demonstrate a definitive convergence. This outcome implies that the size of the available experimental data is a limiting factor and suggests that the performance of the ML model is likely to continue improving with the addition of more training data. That said, we chose not to increase the training size beyond the aforementioned train-to-test split ratio, as doing so would diminish the size of the test set, potentially compromising the thoroughness of the evaluation of our ML model.

#### 3.1.3 Feature Interpretation

In the classification analysis, our GBFS workflow identified the range of periodic table column (or group) numbers among the elements in a chemical composition as the most salient feature. Upon analyzing the relationship between this feature and the median *T*_c_ values in the training set, we observed a correlation coefficient of around 0.52, where higher values of this feature generally correspond to higher *T*_c_ values. For instance, a range of periodic table column numbers less than 10 is typically associated with superconductors having a median *T*_c_ below 15 K. Conversely, we observe that the median *T*_c_ exceeds 40 K when the feature value surpasses 10. The peak of the feature distribution (i.e., the highest median *T*_c_ value) is observed when the range of periodic table column numbers is 14. This result is consistent with findings from other research, including a notable study by Stanev et al.,^26^ which identified the standard deviation of the periodic table column number as the most relevant predictor in their classification analysis. This finding is complemented by the importance of statistical measures related to the electronegativity, melting temperature, and atomic weight of the elements comprising the composition.

A possible interpretation appears to be associated with the influence of the observed variations in electron configuration and bonding characteristics on the physical properties of materials. For instance, elements with a single valence electron, such as alkali metals, exhibit distinct properties from transition metals, which often have partially filled *d*-orbitals. These *d*-orbitals contribute unique electronic characteristics that can be crucial for superconductivity. Furthermore, a wide range of the periodic table column numbers of the elements in a compound’s formulation suggests high diversity in their electronic properties. This high diversity appears to be pivotal, as superconductivity is intricately linked to the electronic structure of the material. Elements from various groups influence the band structure differently, thereby affecting the material’s conductive properties. Consequently, a broader range in column numbers not only signifies greater chemical complexity but may also indicate a more complex electronic band structure, which can be favorable for achieving higher *T*_c_, as we will discuss later. This high level of complexity can enhance the superconducting properties of materials, making the range of periodic table column numbers a significant predictor in superconductivity studies. A clear example can be observed via cuprate materials, which have complex compositions and an extensive variety of constituent elements, making them likely to exhibit a broader range in this feature.

The second salient feature was identified as minimum molar volume. The molar volume of a material, which measures the volume occupied by one mole of a substance, appears to be a key feature for predicting the *T*_c_ of superconductors due to several reasons related to the material’s structural and electronic properties.

Molar volume impacts the electron–phonon coupling in a material, an important mechanism for superconductivity. In superconductors, the atomic spacing affects phonon modes—quantized modes of vibration within the crystal lattice that are essential for the electron pairing mechanism. This interaction is particularly crucial in conventional superconductors, where electron pairs (i.e., Cooper pairs) are mediated by phonon interactions. The Debye frequency of phonons underpins the well-established relationship , where *m* is the ionic mass.^41−43^ An increase in molar volume typically corresponds to larger interatomic distances, which can influence both the density and mobility of electrons. The spatial expansion affects the electron–phonon interaction, hindering the ability of electrons to pair and form Cooper pairs, an essential process for achieving superconductivity. The role of electron–phonon coupling and electron correlation strengths in superconductivity is supported by the experimental discovery of the isotope effect,^41,44^ providing strong empirical confirmation for these mechanisms, while the BCS (Bardeen-Cooper-Schrieffer) theory of superconductivity that explains the isotopic effect expresses the superconducting critical temperature as
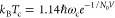
9where *k*_B_ is the Boltzmann constant, *ℏ* is the reduced Planck constant, ω_c_ is a cutoff frequency, *N*_0_ is the electronic density of states near the Fermi level, and *V* is the electron–phonon coupling potential.^2^

Molar volume also relates directly to the lattice constants of a material, which influences the material’s electronic band structure. Changes in the band structure can affect the density of states at the Fermi level (eq 9), a relevant factor for the superconducting properties of materials. Moreover, variations in molar volume affect the internal chemical pressures within a material. This can affect the hybridization of electronic orbitals and thus influence superconductivity. In high-*T*_c_ superconductors, such as cuprates and iron-based superconductors, the internal pressure can modify the electronic structure, thereby affecting *T*_c_. It is well-established that external pressure often elevates *T*_c_ in some superconducting materials; similarly, internal pressures induced by changes in molar volume can exert a comparable effect. By reducing the molar volume (i.e., increasing pressure) in some chemical compounds, superconductivity was observed in a number of high-pressure phases even when the chemical compound was not superconducting at ambient pressure.^45^ This phenomenon is corroborated by empirical data showing that lower values of minimum molar volume are somewhat associated with higher median *T*_c_ values.

The third prominent feature contributing significantly to the reduction in loss within our model is the average deviation of melting temperature among constituent elements in a chemical composition. The melting temperature appears to be an important feature for predicting the *T*_c_ of superconductors due to several interconnected physical and chemical properties.

The melting temperature is indicative of the strength of atomic bonds within a material, and variations in this temperature can influence electron density and mobility. The strength of these bonds affects the dynamics of electrons and phonons, which are fundamental to the mechanisms of superconductivity, particularly in conventional superconductors where electron pairing is mediated by phonon interactions. Consequently, disparities in melting temperatures can create varied local electronic environments, leading to a spectrum of phonon behaviors within the material. These variations, in turn, impact the electron–phonon interactions that are fundamental to the mechanism of superconductivity, as articulated by the BCS theory of superconductivity (eq 9).

Furthermore, the analysis indicates that variations in melting temperature among the constituent elements correlate with the median *T*_c_ value up to a certain value. This indicates a possible enhancement of lattice vibrations that may facilitate the formation of Cooper pairs. Nevertheless, this trend reverses as the distribution of this feature exhibits a broad peak around 500 K. Such a pattern implies an optimal standard deviation in the melting temperatures among the constituent elements, which appears to maximize the positive influence on *T*_c_ predictions. Alternatively, a high standard deviation in melting temperatures may be interpreted as an indicator of considerable variability in the thermal stability of the constituent elements. Such variability could imply a lack of homogeneity in the material’s composition, potentially leading to inhomogeneities in local electronic and phononic properties. These disparities across different regions of the material may influence its overall capability to transition into a superconducting state. The findings suggest that there exists an optimal value for melting temperature variability, which could maximize the material’s superconducting potential. This observation indicates a complex relationship between melting temperature and superconductivity.

It is necessary to recognize that due to the nonlinear characteristics of our ML model, the likelihood of a chemical composition exhibiting a *T*_c_ value greater than 10 K is greatly influenced by the interaction among various features, some of which may exhibit notable correlations. Consequently, these interactions can result in a distinct feature profile, differing from those observed in other studies. The complex nature of this matter mandates careful consideration and an analysis of feature interdependencies within the predictive modeling framework. Our examination of these features emphasizes the intricate relationships that govern superconductivity and further accentuates the multifaceted nature of predictive modeling in superconductor research.

### 3.2 Regression Analysis of Critical Temperatures

#### 3.2.1 Overall Performance Evaluation

Having established an effective classification model, we now advance to the more intricate task of developing a regression model to predict the critical temperatures (*T*_c_) of superconductors based on their chemical composition alone. This endeavor is considered more complex because it involves quantifying the magnitude of *T*_c_, rather than merely categorizing compounds based on their potential to exhibit discernible superconducting properties. Specifically, the development of a regression model not only deepens our understanding of the factors influencing *T*_c_ in known superconductors but also serves as a crucial element in initiatives aimed at discovering new superconducting materials.

We employed the same set of features that were used to create the classification model to formulate four distinct regression models. Initially, a ML model was trained on the entire training data set using our GBFS workflow, which we will refer to as the ‘all-*T*_c_’ model. In addition, we tailored specific regression models for distinct families of superconductors, namely cuprates and iron-based superconductors. A fourth ML model was then developed to handle data that did not fall into these categories, which we will refer to as ‘low-*T*_c_’ group owing to their relatively smaller *T*_c_ values. We exclusively considered superconductors with valid *T*_c_ records, where we computed the median *T*_c_ values for duplicated chemical entries, and employed a random splitting methodology to divide the data. We maintained an 85%:15% train-to-test split ratio and strictly trained the model to be agnostic to crystalline polymorphs. This approach enables modeling without the requirement for 3-D crystallographic data, thereby permitting a methodical analysis across a wide set of superconducting materials.

**Figure 8:**
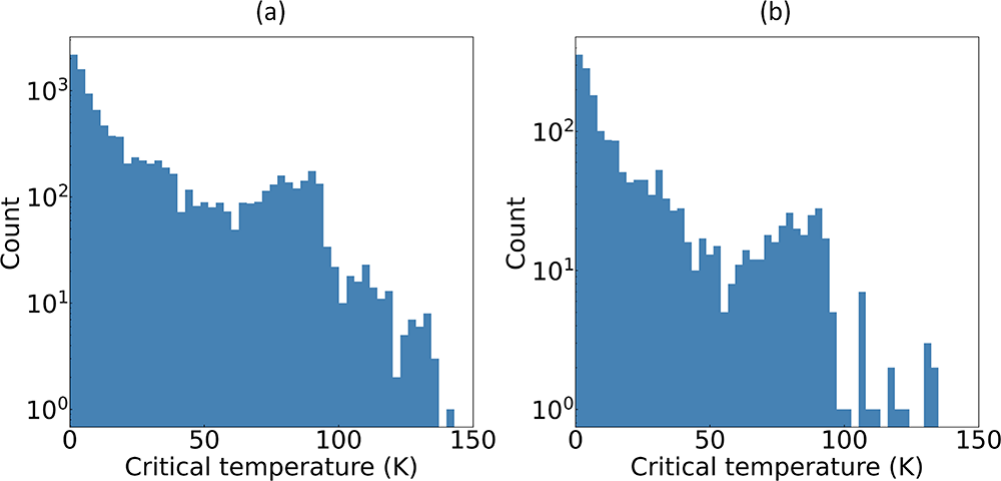
Distribution of superconducting critical temperature (*T*_c_) values in (a) the training set and (b) the test set.

We begin our analysis with a performance evaluation of the all-*T*_c_ regression model. The results of the random data partitioning are depicted in Figure 8, which displays the distribution of *T*_c_ values among known superconductors. Notably, the majority of the *T*_c_ records are concentrated below approximately 50 K, with a rapid decline in the frequency of superconductors at higher *T*_c_ values. These distributions highlight the skewed nature of *T*_c_ within the data set, which has been complied from the scientific literature.

**Figure 9:**
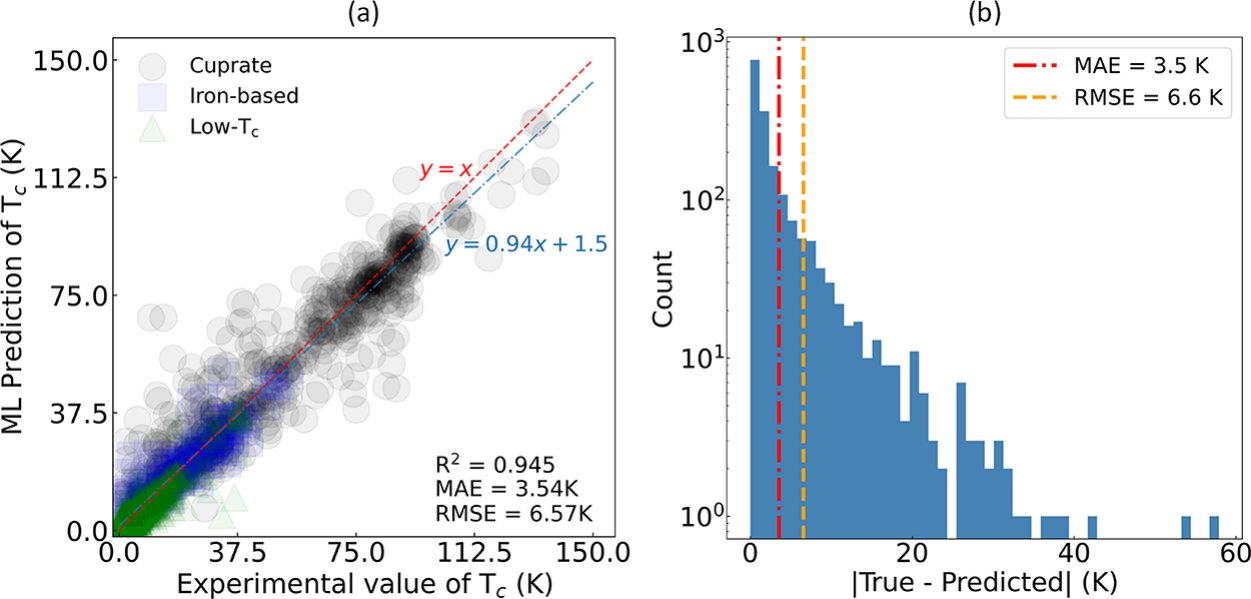
(a) ML-based predictions of critical temperature (*T*_c_) plotted against the experimental values (ground truth) for the regression model that has been trained and Bayesian-optimized on the final subset of features selected by our GBFS workflow. Dashed red line illustrates the hypothetical scenario in which the ML-based predictions perfectly match the experimental values. Blue dot-dash line represents a linear fit produced via the Ordinary Least Squares (OLS) method. Scatter plot is color-coded to represent the different types of superconductors, where black circles correspond to cuprate, blue squares to iron-based, and green triangle to low-*T*_c_ superconductors. Accompanying panel in (b) displays the distribution of absolute errors in the predictions, where the dashed red line (−.) signifies the MAE and the dashed orange line (−−) represents the RMSE. These results pertain to the out-of-sample test set.

Figure 9a presents the ML-based predictions of *T*_c_ plotted against experimental values that has been generated by the all-*T*_c_ model. This model is based on a Bayesian-optimized gradient boosting algorithm that was refined using 34 features selected from an initial set of about 730 exploratory features via our GBFS workflow. The blue dot-dash line represents the line of best fit, established through the Ordinary Least Squares (OLS) method. This line illustrates the relationship between our ML-based predictions and the ground truth. The linear fit has a gradient of 0.94 and a *y*-intercept of 1.5 K. The *y*-intercept of the linear fit indicates a small systematic bias for lower *T*_c_ values, while the gradient reveals an almost ideal alignment between our model’s predictions and the experimental measurements, albeit with a minor underestimation at higher temperatures within the examined range.

The apparent deviations in model predictions at higher temperatures are understandable, considering the predominant concentration of data within the lower temperature range. The reduced data density at higher temperatures likely contributes to the discrepancies between the ML predictions and the ground truth within this range, as evidenced by the line of best fit (blue) in Figure 9a, which is positioned below the diagonal lines (red). Despite these challenges and the absence of any 3-D structural information, our ML approach has consistently provided accurate predictions of *T*_c_, demonstrating its utility in modeling superconductors across varied conditions. Please refer to Supporting Information 2, where these results have been recreated, categorized by the different types of superconductors. With a closer examination of the error distribution plots presented in Figure 9b, it is evident that the majority of the prediction errors are below 10 K. Further analysis of the chemical compositions within the test set reveals that approximately 91% of cases exhibit an absolute error below 10 K, about 79% display errors below 5 K, and around 39% maintain errors below 1 K.

We observed that 13 chemical compositions out of approximately 1800 in the test set exhibited deviations greater than 30 K between predicted and experimental values. These compounds predominantly belong to various families of cuprate superconductors which are known for their layered crystal structures that incorporate copper-oxide (Cu–O) planes; these being critical for their superconducting properties. These include YBCO (Yttrium Barium Copper Oxide), where yttrium may be substituted by other elements; BSCCO (Bismuth Strontium Calcium Copper Oxide), characterized by their phase diversity and varying numbers of copper oxide planes; TBCCO (Thallium Barium Calcium Copper Oxide) and Tl-based compounds, where thallium substitutes bismuth; mercury-based superconductors, noted for achieving high critical temperatures; and lanthanum-based superconductors, which involve complex substitutions affecting their superconducting characteristics. This finding highlights the intricate relationship between chemical composition and superconducting properties in these superconductors.

These results are further substantiated by an *R*^2^ of 0.945, an MAE of 3.54 K and an RMSE of 6.57 K, reflecting a strong correlation between predictions and experimental measurements. The higher RMSE reflects greater penalization for predictions that deviate from their true values. The distribution of absolute errors is shown in Figure 9b, further detailing the performance nuances of this predictive model. During a 10-fold cross-validation procedure, the model afforded an *R*^2^ of 0.94 ± 0.01, an MAE of 3.73 K ± 0.13 K, and an RMSE of 7.45 K ± 0.65 K. These metrics highlight the model’s consistent predictive accuracy and reliability across multiple subsets of the data. We attribute the performance result to the implementation of our GBFS workflow, which effectively minimized feature redundancy and maximized relevance to the target variable. The efficacy of our modeling strategy enabled us to surpass the performance of other studies by employing a systematic approach to feature analysis and selection, and notably, without resorting to the use of regularization techniques during model optimization. The results on both the test set and the cross-validation process further affirm the generalizability of our model, without overfitting to the training set.

To rigorously evaluate the results of our study against those obtained from other methods that were discussed in the Section 1, we have compiled a table that encapsulates key performance metrics from various studies. Table 2 presents a comparative analysis of test-set results from diverse ML algorithms that were used to predict superconductivity in materials, examining evaluation metrics such as *R*^2^, MAE, RMSE, Accuracy, F1-Score, and AUC-ROC. This comparison delineates the distinctions and parallels in model performance, providing insights into how our study aligns in terms of accuracy, generalizability, and predictive precision.

**Table 2:** Summary of Test-Set Results for the Regression and Classification Analyses Using Different ML Methods to Predict Superconductivity of Materials

algorithm	train-to-test split	*R*^2^	MAE (K)	RMSE (K)	accuracy	F1-score	AUC-ROC
GBFS (this study)	85%:15%	0.945	3.54	6.57	0.947	0.912	0.94
random forest^26,27^	85%:15%	0.885			0.92	0.92	
deep-CNN^a^^28^	95%:5%	0.92			0.95	0.77	0.94
ensemble DeepSet^a^^30,31^	80%:20%	0.93		9	0.84	0.71	

a Indicates that the corresponding classification analysis incorporated a test set from an additional source, specifically ref (29).

The findings confirm that our GBFS-based model, employing a train-to-test split of 85%:15%, achieves superior performance with an *R*^2^ of 0.945, signifying its ability to explain 94.5% of the variance within the test set. Furthermore, this model exhibits robust classification capabilities, which indicates a well-balanced precision and recall and illustrates its proficient discrimination capacity. The random-forest model, with an identical train-to-test ratio, displays a substantial *R*^2^ of 0.885 and consistent classification performance with both accuracy and F1-score at 92%, albeit slightly lower than that of the GBFS model.

In comparison to deep-learning methodologies such as the Deep-CNN model, which operates with a tighter train-to-test ratio of 95%:5%, it is observed that its regression capabilities are nearly on par with the GBFS-based model, achieving an *R*^2^ of 0.92. Additionally, the DeepSet model, with a train-to-test ratio of 80%:20%, also demonstrates excellent regression performance with an *R*^2^ of 0.93. These results underscore the capability of our GBFS-based model to achieve excellent outcomes, even when juxtaposed with more complex deep-learning approaches. It should be highlighted that the classification results associated with deep-learning methods included a test set from an additional source,^29^ thereby precluding a direct comparison. Moreover, the Deep-CNN model employed a unique testing scheme, specifically the temporal separation testing scheme, as previously mentioned in the Section 1.

Having established that a regression model trained on a comprehensive data set encompassing all types of superconductors performs robustly, we now aim to investigate the impact of training and testing the model on specific groups of superconductors. By adhering to the same modeling approach via our GBFS workflow, we demonstrate the adaptability of our methodology. Such targeted analysis not only tests the flexibility of the modeling technique but also provides insights into how prediction outcomes vary among different superconductor families, which is attributed to the distinct mechanisms that drive superconductivity in each group. This nuanced approach helps to understand the differential behavior and underlying materials-property characteristics that are specific to each type of superconductor.

**Figure 10:**
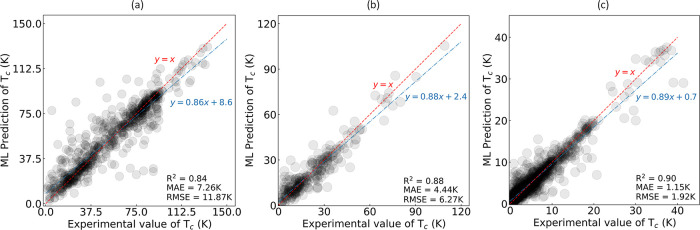
ML-based predictions of critical temperature (*T*_c_) plotted against experimental values (ground truth) in a series of regression models, each trained on distinct groups of superconducting materials: (a) cuprate, (b) iron-based, and (c) low-*T*_c_ superconductors. Each model’s test set is confined to the respective superconducting group upon which it was trained. Dashed red line depicts the ideal scenario where ML-based predictions align perfectly with the experimental values. Blue dot-dash line indicates a linear fit generated using the Ordinary Least Squares (OLS) method. These results pertain to the out-of-sample test set.

**Figure 11:**
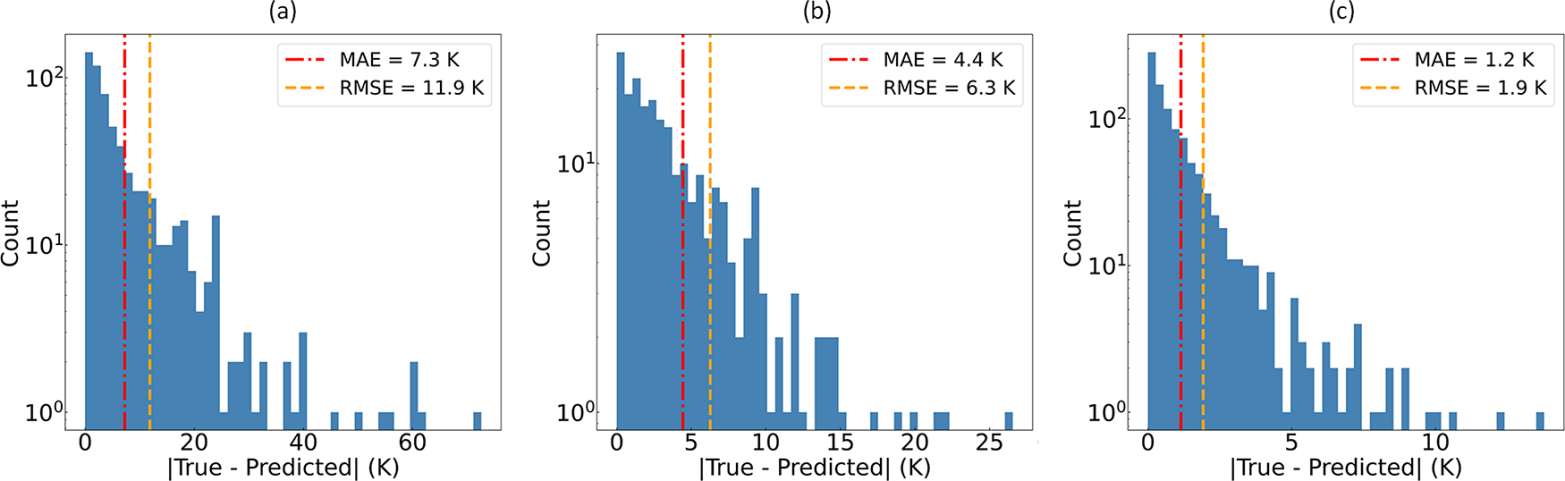
Distribution of absolute errors in the ML predictions of critical temperature (*T*_c_), where each model is trained on distinct groups of superconducting materials: (a) cuprate, (b) iron-based, and (c) low-*T*_c_ superconductors. Dashed red line (−.) signifies the MAE and the dashed orange line (−−) represents the RMSE. These results pertain to the out-of-sample test set.

The high performance of our regression models is consistently observed across different groups of superconducting materials, specifically when models are trained separately on (a) cuprate, (b) iron-based, and (c) low-*T*_c_ superconductors. The results of these models are shown in Figure 10, and the corresponding distributions of absolute errors are depicted in Figure 11.

The performance of our regression models varies by the type of superconducting material used for training. The model based on cuprate superconductors achieved the lowest *R*^2^ value of 0.84, accompanied by an MAE of 7.26 K and an RMSE of 11.87 K. In comparison, the iron-based model demonstrated slightly lower errors, with an MAE of 4.44 K, an RMSE of 6.27 K, and an *R*^2^ of 0.88. Meanwhile, the model trained on low-*T*_c_ superconductors, which constitutes the larger data set with approximately 6600 unique chemical compositions, showed the strongest performance with an *R*^2^ of 0.90, an MAE of 1.15 K, and an RMSE of 1.92 K. This outcome is unsurprising given that the low-*T*_c_ model not only has the benefit of a larger data set but also targets superconductors within a relatively smaller *T*_c_ range. Conversely, the model based on cuprate superconductors is trained using approximately 4,000 unique chemical compositions, with the test set encompassing a temperature range that extends up to 150 K. Meanwhile, the iron-based model is trained on a data set of around 1500 unique compositions, covering a temperature range that falls between those of the low-*T*_c_ and cuprate models.

It is noteworthy that the regression models tailored to specific families of superconductors do not outperform the general model that incorporates all superconductivity data. This observation can be attributed primarily to a compositional effect, whereby each group of superconductors predominantly contributes to a unique range of *T*_c_ values. The composition effect denotes the portion of the observed intergroup variance in the distribution of the target variable (i.e., *T*_c_) that can be attributed to disparities in the distribution of covariates among the groups. This phenomenon may arise from the data set containing multiple groups, each with distinct characteristics, thereby influencing the overall analytical results due to the unique attributes of each group. Consequently, the aggregate regression model, which encompasses a broader spectrum of data across extended temperature intervals, is more effectively calibrated and yields a more accurate determination of *T*_c_ values. As we will see later in Section 3.2.3, each family specific model employs a distinct profile of features for making *T*_c_ predictions. This diversity in feature utilization highlights the unique characteristics of each superconductor family and enables a detailed analysis of model efficacy within each specific material group.

#### 3.2.2 Gradient-Boosted and Statistical Feature-Selection Workflow

Having illustrated the utility of our GBFS workflow in the classification task, we now extend its application to a regression task. While a comprehensive analysis was previously detailed for the classification task, here we only summarize the principal conclusions pertinent to predicting *T*_c_ in order to be succinct; nevertheless, we will describe any significant distinctions when adapting the workflow to a regression analysis.

**Figure 12:**
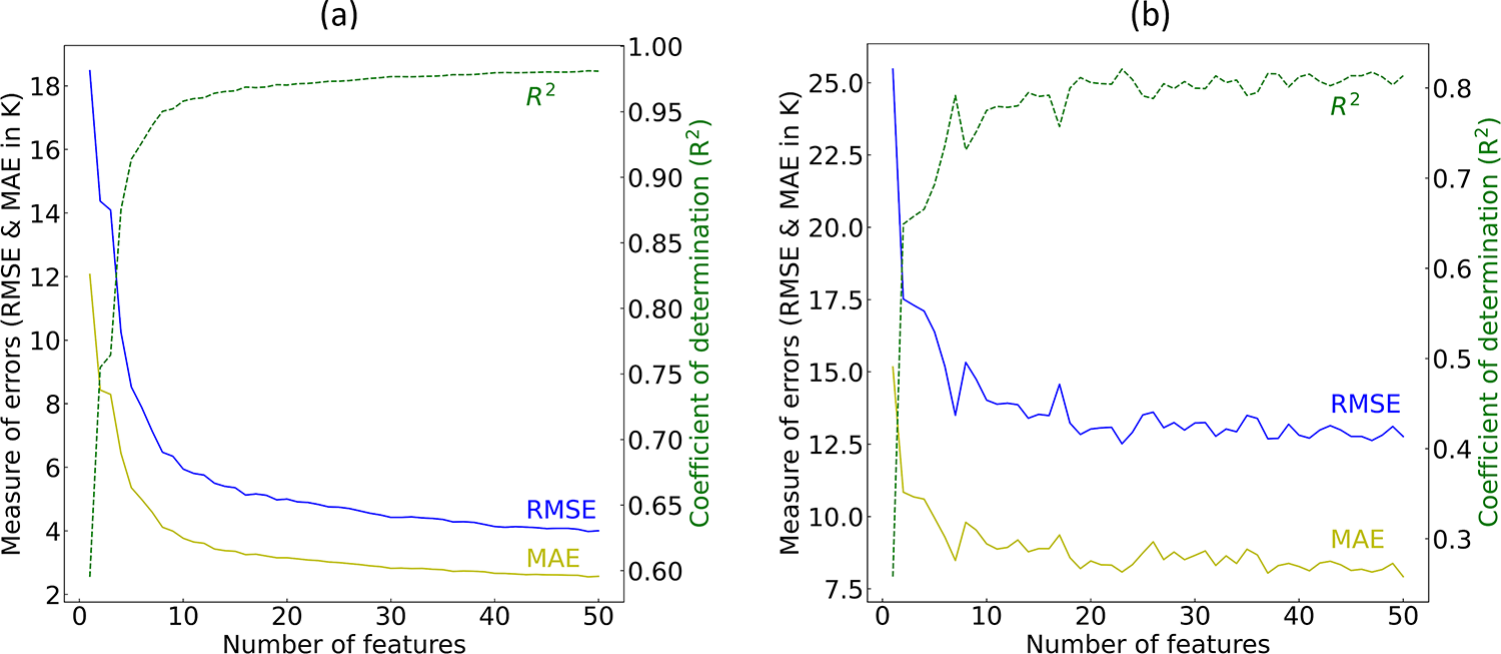
Result of the gradient-boosting feature-selection process in the regression analysis for predicting *T*_c_ values. Performance of GBDTs was assessed on (a) the training set and (b) the validation set, where GBDTs were recursively trained, incorporating an incrementally increasing subset of features, starting with the most relevant feature identified by the greatest total loss reduction observed.

Our GBFS workflow narrowed down an extensive set of around 730 exploratory features and 150 engineered features to a targeted subset of 34 features. This refinement was achieved through the recursive training of GBDTs, optimizing model performance as evidenced by the convergence of key statistical metrics including MAE, RMSE, and *R*^2^. The preliminary ranking of feature relevance was determined based on the aggregate loss reduction observed during the training phase. This process is graphically depicted in Figure 12, which shows that performance metrics plateau after the inclusion of approximately 30 salient features in both training and validation sets. As expected, the trajectory of convergence for the validation set exhibited more pronounced fluctuations, reflecting the typical variability associated with out-of-sample data. It is important to reiterate that at this phase, interdependencies among the features, such as multicollinearity, were not explicitly addressed. Some of the salient features identified at this stage of the workflow include the range of thermal conductivity and the standard deviation of molar volume among the constituent elements within a chemical composition.

A suite of feature analysis ran in parallel to the recursive training of GBDTs, some of which are distinct from those used in our classification study. For instance, in the regression analysis, we applied hypothesis-based testing methods in a bivariate format to explore the causal relationships between exploratory features and the target variable. We specifically employed the F-test for comparing means within the framework of a one-way ANOVA. This approach included evaluating the correlation between two continuous features via the correlation coefficient, *R*, which was subsequently converted into a regression F-statistic as a part of the ANOVA-based regression analysis. These hypothesis-testing techniques were instrumental in facilitating statistical inference, enabling us to ascertain the statistical significance of exploratory features. This was achieved by analyzing the test statistics that were generated from hypothesizing correlations between pairs of features. For example, the linear association between each continuous exploratory feature and the target variable was assessed using a normalized F-statistic for relative comparison. This analysis identified that the feature exhibiting the strongest linear association with the target variable corresponds to the presence of copper and oxygen, as well as statistical measures related to electronegativity among the constituent elements within a chemical composition.

MI analysis revealed that the greatest gain in entropy, or information, about *T*_c_ was obtained by knowing the features that are associated with statistical measures of the boiling point, molar volume, and first ionization energy of the elements within the chemical composition. To quantify this, we employed an MI estimator that employs entropy estimations that are derived from K-nearest neighbor distances. Akin to the classification task, these results guided the creation of additional features. Using a brute-force approach, we derived an additional 156 features, culminating in a preliminary set of 206 features. This enriched feature set is now prepared for extensive regression analysis, facilitated through subsequent stages of our GBFS workflow.

Progressing through the stages of our GBFS workflow, we addressed multicollinearity reduction, evaluated the permutation importance of the refined features, and implemented recursive-feature elimination. These steps culminated in the identification of the optimal subset of features, which were then used for Bayesian optimization of our regression model. This systematic approach ensures that the most impactful features are selected to enhance model accuracy and efficiency, while eliminating potential human bias during model development.

**Figure 13:**
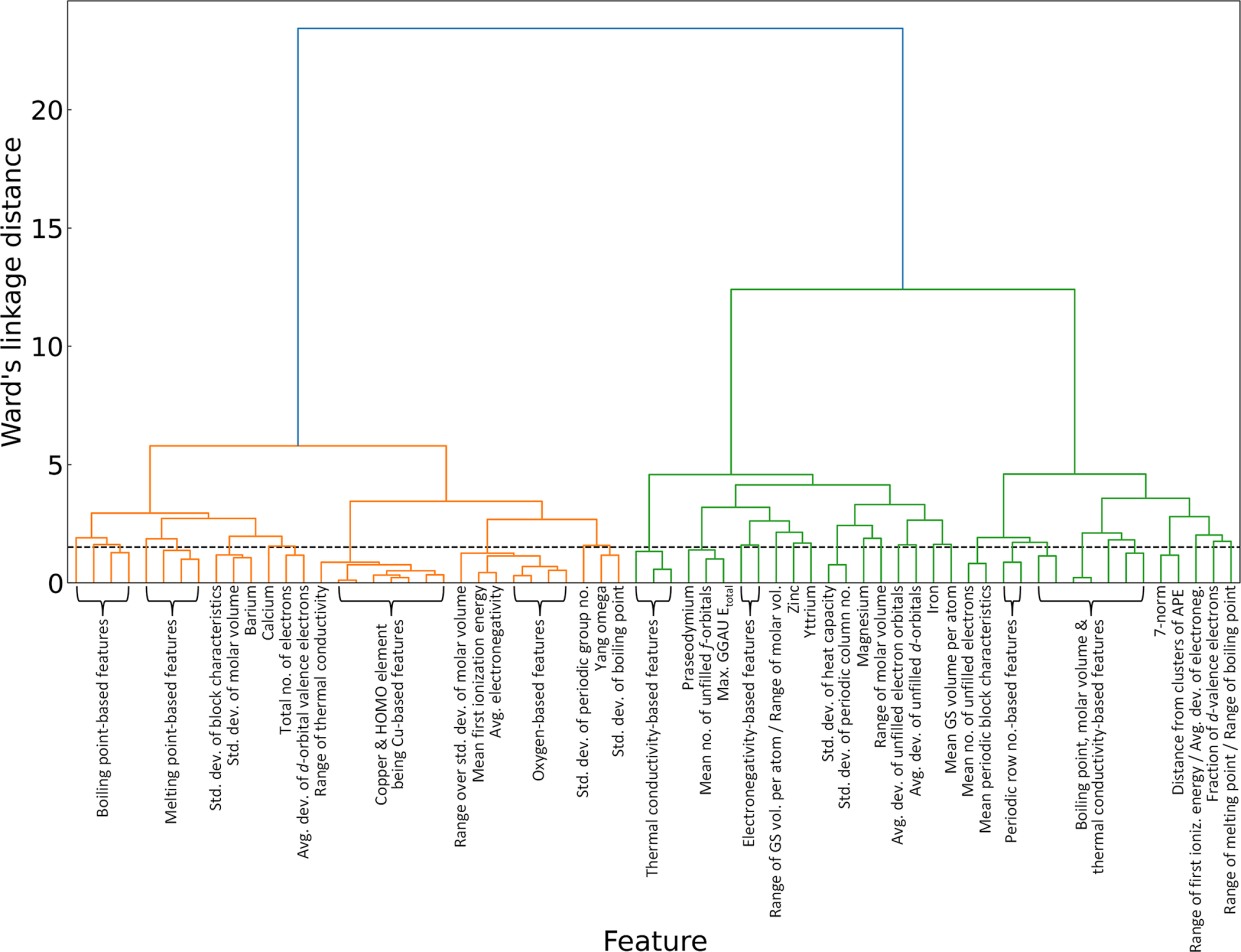
Dendrogram illustrating the results of hierarchical-agglomerative clustering, conducted on the remaining 67 features after applying the correlation analysis. Dashed horizontal line in the dendrogram signifies the established distance threshold of 1.5 units according to Ward’s linkage criterion.

**Figure 14:**
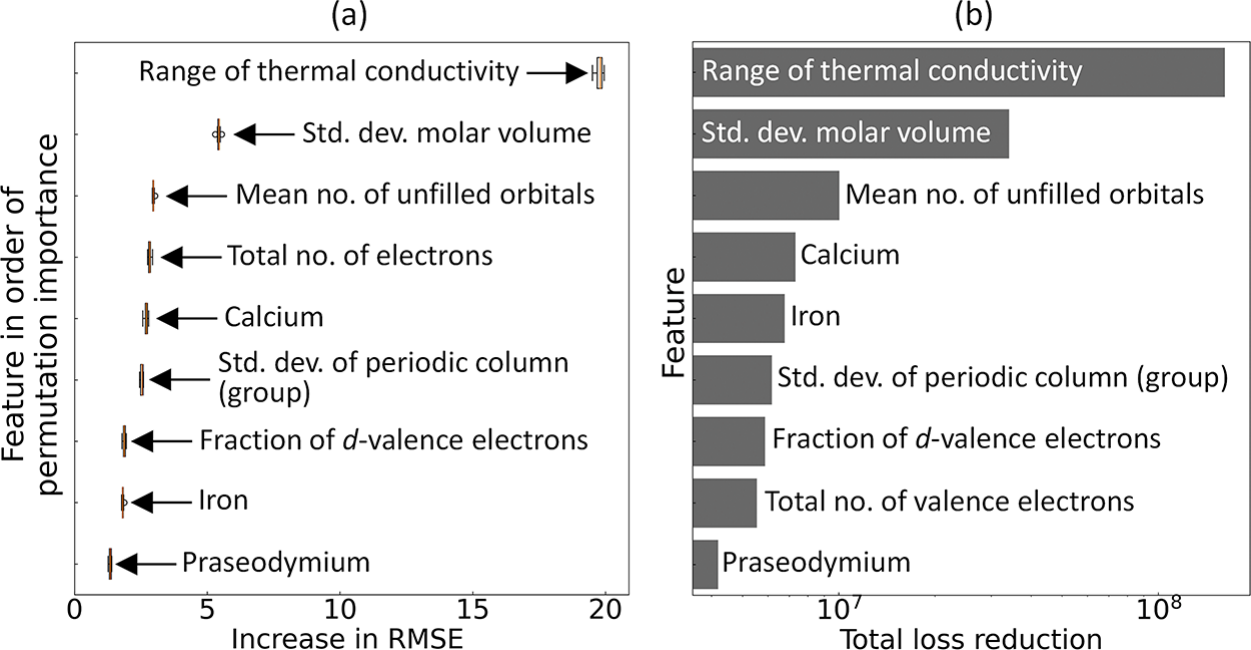
(a) Permutation-based feature importance and (b) feature relevance plots for the regression analysis of *T*_c_. Figure presents the plots illustrating the nine most significant features.

The two-step multicollinearity treatment systematically removed 170 features. Specifically, the correlation analysis eliminated 139 features whose correlation coefficient exceeded 0.8, while the hierarchical-cluster analysis eliminated further 31 features, using the Spearman rank-order correlation with a Ward’s linkage distance threshold of 1.5 units. This curation retained a subset of 36 features. The selection of the optimal distance threshold was informed by the Elbow method, and the resulting dendrogram, which visualizes the hierarchical-agglomerative clustering, is displayed in Figure 13. Furthermore, insights from the 10-fold permutation feature-importance evaluation, illustrated in Figure 14a, emphasize statistical measures associated with thermal conductivity, molar volume, number of unfilled orbitals among constituent elements in the chemical composition as some of the most important features, presented in descending order of their permutation importance.

**Figure 15:**
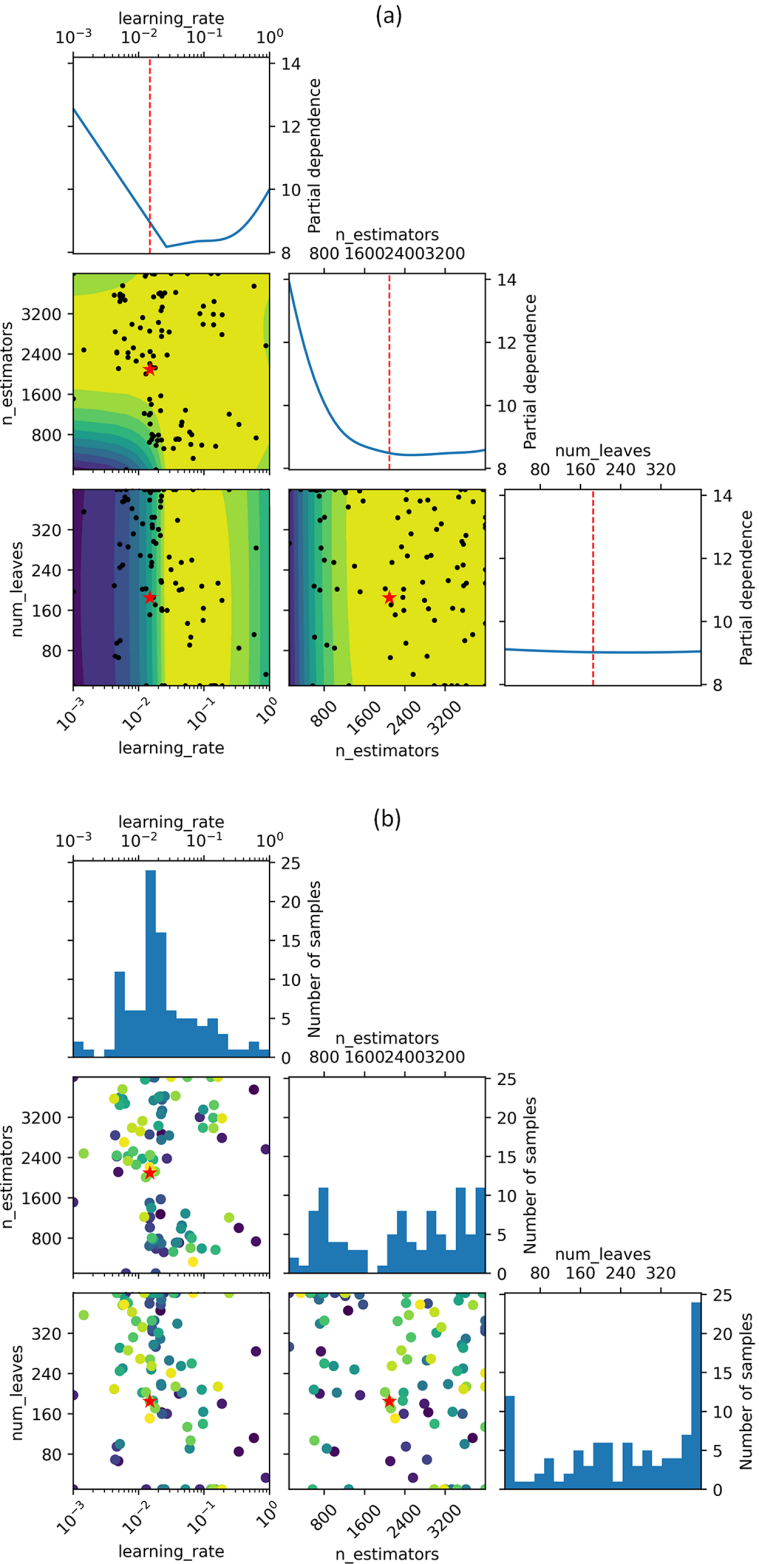
Results of Bayesian optimization for the final regression model using the training data, where (a) depicts the partial dependence plot and (b) illustrates the evaluation plot. Red stars mark the hyperparameter values that correspond to the minimum of the objective function. Dashed vertical red lines indicate the approximate location of this objective minimum, providing a visual guide to the most effective hyperparameter settings within the model’s configuration.

**Figure 16:**
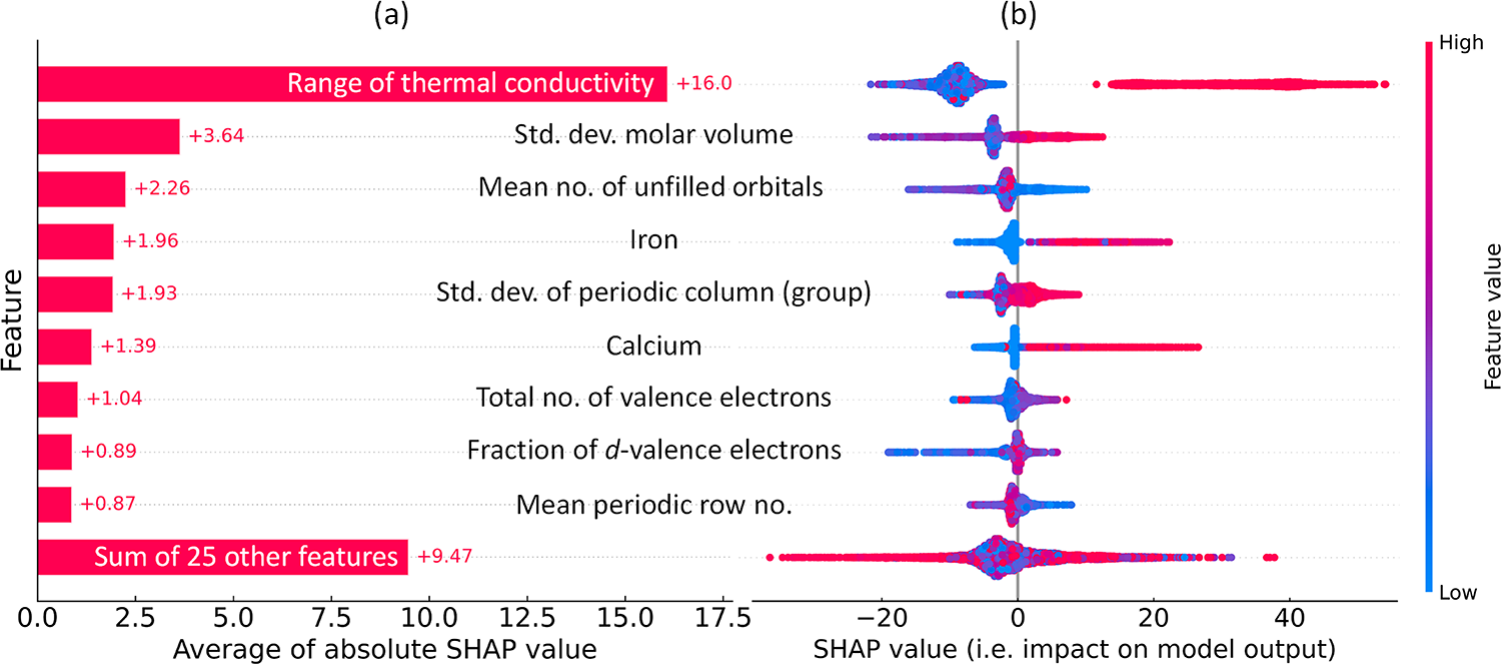
Result generated using the SHAP framework. (a) Average contribution plot, represented by the mean absolute SHAP value, of the nine features identified as having the most significant impact on the model’s output. Positive SHAP value indicates a positive contribution to the regression of *T*_c_. (b) Beeswarm plot visualizes the effect of these features on the model’s output by displaying each data instance as a single point alongside its SHAP value on the *x*-axis, while alignment on the *y*-axis corresponds to the features in (a). Color scheme of each point reflects the original feature value, and the broadening shows the density of instances.

Next, recursive-feature elimination was conducted using a 10-fold cross-validation method with RMSE as the evaluation criterion, refining the feature pool to a subset of 34 features. This final selection was meticulously narrowed down from an initial pool of approximately 730 candidate features, identifying those most salient to the predictive objective without any prior domain-specific knowledge. To finalize our workflow sequence, we implemented the optimization procedures outlined in our classification study to determine the architecture of our final predictive model. Figure 14b illustrates the features that contributed the most to the total loss reduction in the predictions made by the final regression model, while the partial dependence and evaluation plots derived from the Bayesian optimization results are presented in Figure 15. These features align with those identified in earlier phases of the GBFS workflow, and they are corroborated by the results from the SHAP analysis shown in Figure 16. This concurrence reaffirms the consistency and importance of these features across various stages of model development.

In a manner akin to the classification model, two separate regression analyses were undertaken—one excluding the MEGNet embeddings and the other incorporating them. The integration of MEGNet embeddings into the regression framework demonstrated comparable model performance, with two of the embeddings ranking among the top five most relevant features. This consistency underscores their effectiveness in enhancing transfer learning capabilities. Despite their utility, challenges remain in interpreting the specific physical contributions of these embeddings with respect to the final predictions. Therefore, we have chosen to present the results associated with the model that was trained without the MEGNet embeddings, to focus on more interpretable outcomes.

**Figure 17:**
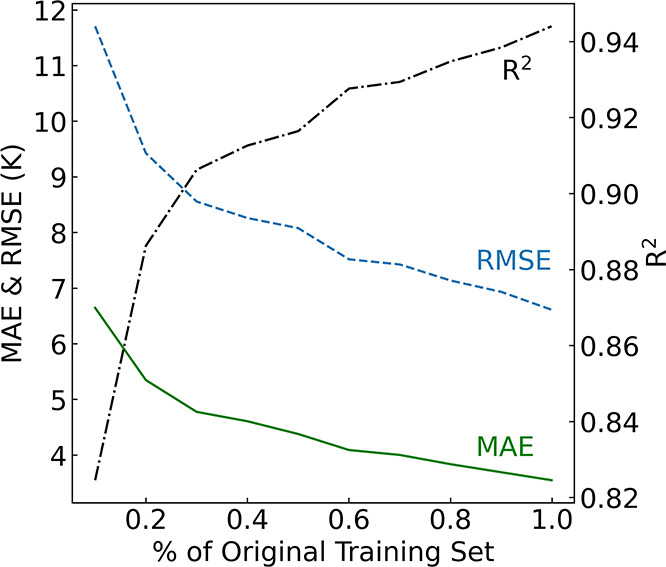
Evaluation of MAE, RMSE, and *R*^2^ conducted by training our ML model on increasingly larger subsets of the original training set, where the data were randomly sampled.

We reexamined the influence of data set size on model performance in our regression analysis to determine if data set size acts as a limiting factor or to pinpoint the optimal training set size. Our analysis involved computing MAE, RMSE, and *R*^2^ for progressively larger subsets of the original training data set, chosen through random sampling. As shown in Figure 17, performance consistently improves as the training set size increases, without a clear convergence of these metrics. These results suggest that the extent of available experimental data may restrict the performance of the model and indicates potential for further improvements with additional data. As with our classification problem, we decided against expanding the training set beyond the existing train-to-test split ratio to prevent diminution of the test-set size, which could compromise the comprehensiveness of the evaluation of our model.

#### 3.2.3 Feature Interpretation

Table 3 summarizes the most salient features that contributed to the results of our regression analyses, including those pertinent to family specific models. We now seek to rationalize their significant role. The foremost influential feature, as denoted by the realized total loss reduction, is the range of thermal conductivity among the constituent elements within the chemical composition. The second most influential feature concerns the standard deviation of molar volume among these elements. Further features demonstrating notable relevance to the target variable include a variety of statistical measures related to the number of unfilled orbitals, the presence of calcium and iron, the periodic column (or group) number, the proportion of *d*-valence electrons, and the total number of electrons. In Section 3.1.3, we examined the significance of molar volume and periodic table column numbers. Consequently, this section will focus on exploring other features that have played pivotal roles in our predictive analysis.

**Table 3:** List of Most Relevant Features Identified by Our GBFS Workflow for the Regression of *T*_c_, Including Subfamily Analyses

feature rank	model type			
	all-*T*_c_	cuprates	iron-based	low-*T*_c_
1	thermal conductivity	no. of unfilled orbitals	periodic column (group)	magnesium
2	molar volume	praseodymium	packing efficiency	space group no.
3	no. of unfilled orbitals	*d*-valence electrons	oxygen	no. of unfilled *p*-orbitals
4	calcium and Iron	calcium	thermal conductivity	niobium
5	periodic column (group)	periodic block	molar volume	first ionization energy
6	*d*-valence electrons	volume per atom	Mendeleev no.	volume per atom

The feature exhibiting the highest relevance in our analysis is the range of thermal conductivity among the constituent elements within the chemical composition. A correlation coefficient of 0.68 between the feature and the target variable (i.e., the median *T*_c_ values) indicates a strong positive trend. This observation is corroborated by SHAP analysis results (Figure 16), which demonstrate that higher values of this feature predominantly correspond to positive SHAP values. There appears to be clear interplay of thermal and electronic properties of superconducting materials that govern this association.

This stands to reason because superconductors are often used in applications that are exposed to high currents or external magnetic fields, whereby efficient heat dissipation is an essential property of the superconducting material due to the substantial heat loads these conditions can generate. High thermal conductivity also helps to maintain the superconductor below its *T*_c_ by effectively dissipating heat generated by electrical resistance or external sources. Materials with higher thermal conductivity are generally more adept at stabilizing their temperature, thereby helping to maintain superconductivity under varying operational conditions. Thermal conductivity in materials is also intricately linked to phonon (lattice vibration) activity. As previously mentioned, phonons mediate electron pairing that is necessary for the superconducting state through electron–phonon interactions. High thermal conductivity generally indicates efficient phonon transport, which can improve electron–phonon coupling and thereby potentially elevating *T*_c_. This relationship is especially significant in conventional superconductors, where, according to the BCS theory, superconductivity is phonon-mediated. The ability of phonons to efficiently transport heat, a direct consequence of high thermal conductivity, can play a crucial role in influencing the dynamics of electron pairing, which is essential for the onset of superconductivity.

Moreover, a considerable variation in thermal conductivity among the constituent elements of a material may indicate distinct phonon behaviors within the substrate. Such variation can create unique or uneven electron–phonon interactions, which could influence the stability and uniformity of the superconducting state. This effect is particularly pronounced in materials where superconductivity is highly sensitive to local phononic environments. Variability in this trait could lead to localized regions where superconductivity might be either suppressed or enhanced, thereby affecting the overall *T*_c_ value of a material. Such heterogeneity is apparent in the empirical data. Empirical analysis of the distribution of this feature reveals multiple distinct peaks in the profile, suggesting the existence of various crystal-lattice environments. These environments, facilitated by conditions related to thermal conductivity, may be conducive to efficient phonon-mediated electron pairing.

Additional factors warranting consideration in the context of thermal conductivity include: low-temperature thermal conductivity, effects of anisotropy, the superconducting order parameter, and the two-fluid model. For instance, at temperatures approaching absolute zero, the thermal conductivity of superconductors tends to be dominated by electron transport. The characteristics of thermal conductivity near *T*_c_ can provide valuable insights into the strength of electron–phonon coupling, which directly impacts *T*_c_. Regarding factors pertinent to experimental observations, anisotropic effects exist in high-*T*_c_ superconductors, such as cuprates. The anisotropy in thermal conductivity, characterized by varying values in different crystallographic directions, can provide information about the anisotropy of the superconducting gap. Additionally, the superconducting order parameter is a central feature of the BCS theory, describing how the electron pairs condense into a coherent quantum state with lower energy than the normal (nonsuperconducting) state. Fluctuations in the superconducting order parameter can influence thermal conductivity near the transition temperature—a phenomenon that is observed in various high-*T*_c_ materials. More specifically, the magnitude of the order parameter corresponds to the energy gap in the electronic structure of a superconductor that forms at the Fermi surface. This gap represents the energy required to break Cooper pairs into individual electrons, thus disrupting the superconducting state. Below *T*_c_, thermal conductivity can also be interpreted using the two-fluid model, which conceptualizes the material as comprising both normal (nonsuperconducting) and superconducting electrons. The proportion of electrons in the superconducting state significantly affects the overall thermal conductivity, as superconducting electrons do not contribute to thermal transport in the same manner as normal electrons. While the relationship between thermal conductivity and *T*_c_ is inherently linked through the material’s thermal and electronic properties, it is modulated by various factors including the material’s structural characteristics, purity, and the nature of superconducting pairing. These factors must be considered to fully understand how *T*_c_ can be optimized or predicted based on thermal conductivity measurements.

Another salient type of feature is associated with the number of unfilled orbitals, the fraction of *d*-valence electrons and the total number of valence electrons, for which a clear rationale exists. The number of unfilled orbitals pertains to electron configurations of constituent elements within a chemical composition. Analysis of the training set reveals a considerable correlation of −0.49 between *T*_c_ and the mean number of unfilled orbitals, with the distribution of the feature peaking at around a value of one. This suggests that an optimal value of one unfilled orbital, on average, is conducive for superconductivity. Transition metals are characterized by multiple unfilled *d* and *f* orbitals, contributing to intricate electronic structures that are advantageous for high-*T*_c_ superconductivity. This characteristic is prominently observed in cuprates and iron-based superconductors. For instance, copper is a key component in cuprate superconductors and it has one unfilled orbital, indicated by its electronic configuration [Ar]4s^1^3d^10^ (or [Ar]4s^2^3d^9^ before forming an oxide).

It is therefore unsurprising that such a feature would be highly relevant for the cuprate-based model, given that structures incorporating multiple superconducting copper oxide (Cu–O) planes per unit cell tend to exhibit higher *T*_c_ values. Specifically, a higher proportion of Cu and O per formula unit correlates with a reduction in the average number of unfilled orbitals—typically two for O and one for Cu. Furthermore, cuprates typically comprise layers of Cu–O structures, which are stabilized through the addition or substitution of cations like Ba^2+^ and La^3+^. These cations possess a large number of unfilled orbitals, thereby elevating the average number of unfilled orbitals within the compound. This suggests that the ability of interlayer cations to supplement charge to the Cu–O planes could be a crucial factor in influencing the superconducting properties of these materials.

The relationship between *d*-valence electrons and the *T*_c_ value for superconductors is complex and is rooted in the electronic structure and behavior of the materials. The corresponding *d*-orbitals tend to be partially filled in transition metals and their compounds. These *d*-orbitals contribute to the density of states at the Fermi level, a key determinant in superconductivity. A heightened density of states at the Fermi level can lead to an increase in the *T*_c_ value. Additionally, *d*-electrons are known for their strong electron–electron interactions, which arise from their close proximity in energy levels and spatial distribution. These interactions are pivotal in facilitating unconventional superconductivity, as commonly seen in high-*T*_c_ superconductors including cuprates and iron-based superconductors. In the realm of conventional superconductors, *d*-electrons can enhance electron–phonon coupling. Such enhanced coupling mediated by *d*-electrons can elevate *T*_c_ via the mechanism described by the BCS theory of superconductivity.

Another consideration may be linked to magnetic fluctuations and crystal-field effects. For example, *d*-orbitals are known to contribute to magnetic fluctuations. In high-*T*_c_ superconductors, such as cuprates and iron-based superconductors, magnetic interactions mediated by *d*-electrons are integral to the electron-pairing mechanism. Moreover, the stabilization of the crystal field in materials containing *d*-valence electrons influences the electronic environment by causing a split in the *d*-orbital energy levels. This splitting alters the electronic band structure, which can affect the superconducting properties of the material.

Empirical studies have frequently demonstrated that materials featuring *d*-valence electrons are capable of achieving higher *T*_c_ values under specific conditions. Notably, materials such as cuprates (which incorporate copper *d*-orbitals) and iron-based superconductors (which involve iron *d*-orbitals) are known for their high *T*_c_ values and intricate phase diagrams. These characteristics are influenced by the properties of their *d*-valence electrons. Furthermore, experimental modifications to the electronic configurations of *d*-valence electrons, achieved through methods such as doping or the application of external pressure, have been effectively employed to optimize and elevate *T*_c_. Our analysis of the training set suggests that the optimal fraction of *d*-valence electrons for superconductivity lies approximately between 0.2 and 0.5. It is also noteworthy to mention that theoretical models that incorporate the behavior of valence electrons, such as resonating valence bond (RVB) theory which builds upon Hubbard and t-J models, are instrumental in enhancing our understanding and prediction of the superconducting properties of these complex, strongly correlated materials.^46−52^ These theoretical models provide insights into the electronic interactions that may govern superconductivity in materials characterized by *d*-valence electrons.

The significance of the number of valence electrons in influencing the *T*_c_ value of superconductors is underscored by an empirical relationship first identified by Matthias in 1955.^53^ Matthias observed a relationship between *T*_c_ and the number of valence electrons per atom, noting that optimal conditions for superconductivity tend to occur at 5 and 7 valence electrons per atom. The study led to a conclusion that a larger number of valence electrons (≳4) can be favorable for the occurrence of superconductivity, although an exception exists for electronic configurations near six valence electrons. This anomaly is characterized by a noticeable decrease in the *T*_c_ value, which is thought to arise from reductions in paramagnetic susceptibility and electronic specific heat. These phenomena are attributed to a reduced effective number of free electrons.

The prominence of chemical elements such as copper and iron in the context of superconductivity are self-explanatory, especially in relation to cuprates and iron-based superconductors which have been highlighted throughout this discussion. Nevertheless, it is noteworthy that features based on calcium have also emerged as prominent in relation to *T*_c_. This observation warrants a more detailed exploration to elucidate the specific role and mechanisms by which calcium influences superconducting properties.

Calcium is commonly found in various superconductors. Examples include cuprate superconductors based on the Bi–Sr–Ca–Cu–O system,^54^ cuprate superconductors based on the Hg–Ba–Ca–Cu–O system,^55−57^ calcium intercalated graphite compounds,^58^ calcium superhydrides,^59^ among many others.^60−62^ In the Supercon database, approximately 14% of the chemical compositions contain calcium, with *T*_c_ values ranging up to about 140 K. Besides, calcium is frequently used as a dopant in superconducting materials. During the doping process, calcium can substitute for other elements within the crystal lattice, thereby modifying the electronic structure and potentially elevating *T*_c_. When introduced into superconductors, particularly in cuprates or iron-based superconductors, it can alter the carrier density within the material. An increase in carrier density then can elevate *T*_c_ by enhancing the superconducting pairing mechanism. Additionally, calcium plays a key role in stabilizing the crystal structure of iron-based superconductors, which is vital for maintaining the pathways necessary for effective electron flow and pairing. The incorporation of calcium can adjust the balance between electron-like and hole-like carriers, an influential factor in the mechanism of superconductivity in these materials.

The final note in this section underscores the distinct feature profiles for each subfamily of superconducting groups, as outlined in Table 3. This differentiation in feature profiles is anticipated given the different underlying material-property mechanisms across these families of superconductors. It is noteworthy to mention that the most relevant features for the cuprate and iron-based models align with those identified by Stanev et al.,^26^ whose modeling approach differs to that employed in this study. These results indicate that our modeling approach effectively captures the distinct characteristic of different families of superconductors, each driven by unique mechanisms of superconductivity. However, the feature profile for the low-*T*_c_ model is different, which is not surprising given that this group was formed by aggregating the residual materials after segregating cuprates and iron-based superconductors. Consequently, the low-*T*_c_ data set lacks a specific mechanism governing superconductivity, as it comprises a heterogeneous mix of various materials. We expect to observe improvements in predictive accuracy for these analyses when incorporating additional features that extend beyond mere chemical composition. Specifically, the incorporation of features derived from crystallographic data, density of states, doping levels, as well as experimental conditions such as external pressure, is expected to refine the prediction accuracy of *T*_c_ values against their experimental measurements. These expanded feature sets are likely to capture more complex interactions and dependencies within these materials, offering a deeper understanding of superconducting behaviors.

### 3.3 Predictive Capabilities for ML-Model Applications

#### 3.3.1 Case Study 1: Analysis of Out-of-Sample *T*_c_ Predictions

The findings reported thus far provide predictions of *T*_c_ values juxtaposed against experimental measurements, yielding promising statistical figures-of-merit. Nonetheless, it is important to validate these results by considering the predictions within a distinct range of temperatures of specific interest, rather than merely demonstrating their collective statistical quality in an anonymized form. Therefore, we conducted a comparative analysis using experimental measurements of high-*T*_c_ superconductors that had not been previously encountered by our ML model during the training phase. Such an analysis allows us to take a closer examination to assess its predictive capability.

The predicted *T*_c_ values were compared against experimental measurements, with the analysis of 50 examples provided in Table 4. These examples were randomly selected from instances where the experimental measurement of a *T*_c_ value exceeded 85 K. This selection criterion is important, as materials displaying superconductivity at elevated temperature (cf. relative to the liquid-nitrogen temperature) are highly desirable for various practical applications.

**Table 4:**
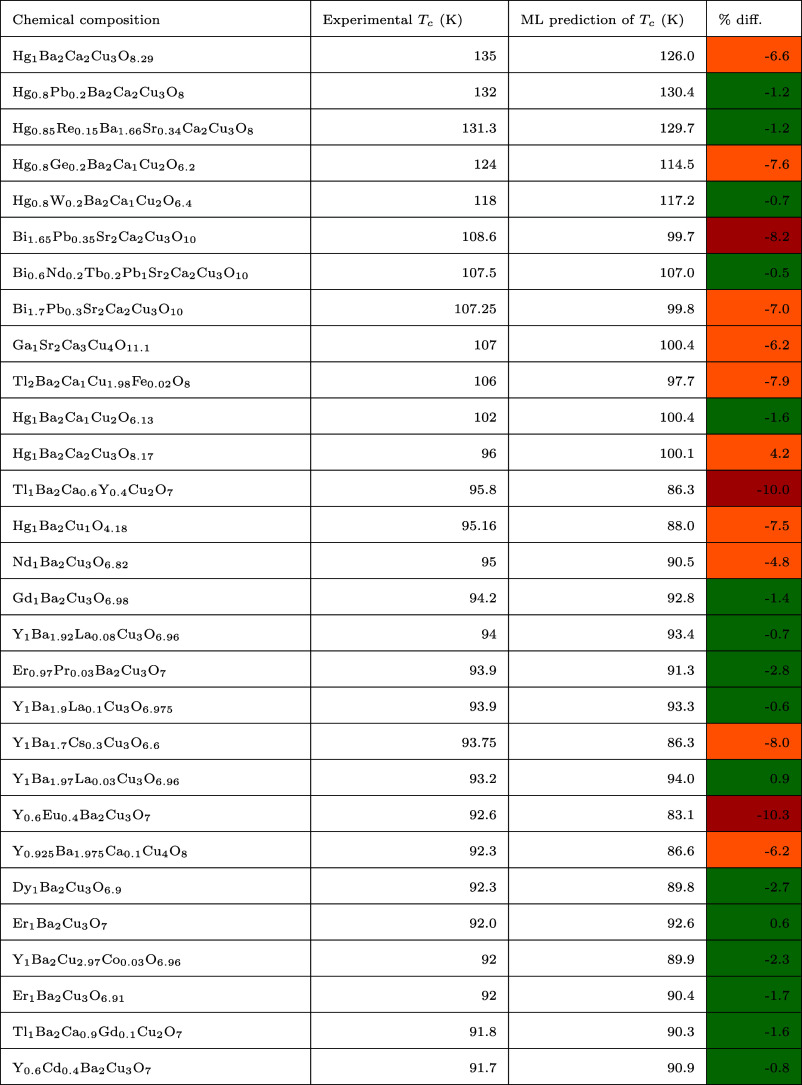

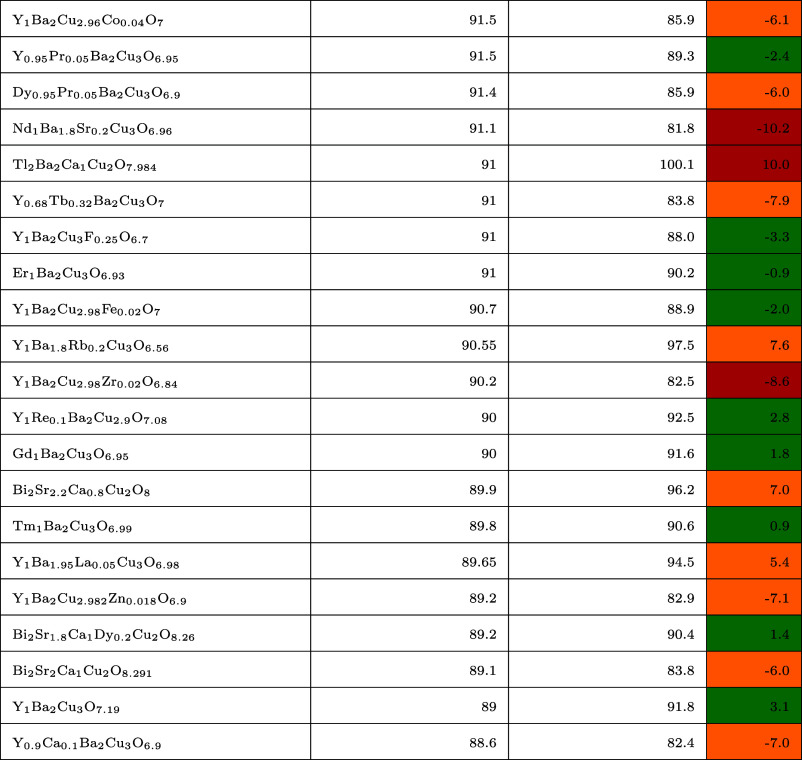
50 Examples of Chemical Composition (i.e., Model Input) and the Corresponding Prediction of Critical Temperature (i.e., Model Output of *T*_c_), Along with the Percentage Difference from the Experimental *T*_c_ Values (i.e., The Ground Truth)^a^

a The results are sorted by the magnitude of *T*_c_ values in descending order. The absolute percentage difference between the predicted values and the experimental measurements are color-coded in green (0–4%), amber (4–8%) or red (>8%).

The propensity of our model to underestimate *T*_c_ values against the experimental measurements, particularly at higher temperature ranges, is clearly observed. This trend is evident in 38 out of 50 cases, where the predictions show a negative percentage difference from the experimental values. This underestimation becomes more apparent at higher *T*_c_ values, as illustrated by the line of best fit (blue) in Figure 9a; this line sits below the diagonal with a gradient of 0.94. We attribute this anomalous pattern to the scarcity of data about high-*T*_c_ superconductors in our training set, as well as in the broader scientific literature. Such scarcity can limit the ability of ML models to effectively learn and predict within the higher temperature range. We anticipate that by augmenting the data set with a greater number of data points that represent these higher temperature ranges, one would significantly improve the predictive accuracy of our model.

Despite the apparent negative bias, which is modest in proportion, our ML model demonstrates strong performance in predicting *T*_c_ values on an absolute scale. The mean absolute percentage difference from the ground truth is approximately 4.5%. Specifically, around 88% of these predictions deviate from the ground truth by less than 8% on the absolute scale, and approximately 54% of the predictions exhibit an absolute deviation of less than 5%. In Table 4, the absolute percentage difference of each predicted *T*_c_ value from its experimental measurement is color-coded according to the classifications: green (0–4%), amber (4–8%) or red (>8%). Only 6 out of the 50 examples were classified in the red category; thereby, demonstrating the efficacy of our ML model in accurately predicting *T*_c_ values. Some of the lowest absolute percentage differences were observed for chemical compounds such as Er_1_Ba_2_Cu_3_O_7_, Bi_0.6_Nd_0.2_Tb_0.2_Pb_1_Sr_2_Ca_2_Cu_3_O_10_, Y_1_Ba_1.9_La_0.1_Cu_3_O_6.975_, and Hg_0.8_W_0.2_Ba_2_Ca_1_Cu_2_O_6.4_. Meanwhile, some of the highest absolute percentage differences were noted for chemical compounds such as Tl_2_Ba_2_Ca_1_Cu_2_O_7.984_, Nd_1_Ba_1.8_Sr_0.2_Cu_3_O_6.96_, and Y_0.6_Eu_0.4_Ba_2_Cu_3_O_7_, whose absolute percentage differences from the ground truth exceed 10%.

#### 3.3.2 Case Study 2: Electronic Density of States of Er_1_Ba_2_Cu_3_O_7_

**Figure 18:**
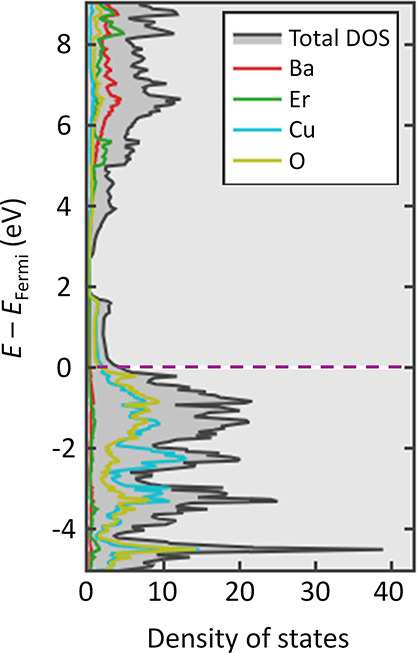
Electronic density of states (DOS) of Er_1_Ba_2_Cu_3_O_7_ derived from density functional theory calculations sourced from the Materials Project.^63^

We now take a closer took at one of the chemical compositions in Table 4, namely Er_1_Ba_2_Cu_3_O_7_. This compound is a type of high-*T*_c_ cuprate superconductor and is part of the generic YBa_2_Cu_3_O_7_ (YBCO) structural family, where yttrium (Y) is substituted by another rare-earth element erbium (Er) in this instance. The orthorhombic crystal structure of this material mirrors that of YBCO and contains Cu–O planes interspersed with layers containing Ba and Er, where the Cu–O planes are essential for superconductivity. The electronic density of states (DOS) for Er_1_Ba_2_Cu_3_O_7_ is depicted in Figure 18. This was derived from density functional theory calculations sourced from the Materials Project.^63^

In the context of superconductivity, a flat band refers to an energy band in which the energy levels remain relatively constant across a range of momentum values, indicating a high degree of electronic-state localization. Such flat bands markedly influence the electronic DOS at particular energy levels. The DOS is inversely proportional to the gradient of the energy-band dispersion. Therefore, in flat bands where the dispersion curve is horizontal (indicative of zero gradient), the DOS becomes singularly large. This explains why the presence of a high DOS at the Fermi level is important for superconductivity, particularly according to the BCS theory, which posits that a higher DOS at the Fermi level increases the likelihood of Cooper pair formation under favorable conditions. In cuprate superconductors such as Er_1_Ba_2_Cu_3_O_7_, the electron pairing mechanism may involve more complex interactions than those characterized by the conventional BCS theory. In such systems, the presence of flat bands just below the Fermi level can enhance these interactions, thereby enhancing electron pairing probabilities. Notably, the DOS for Er_1_Ba_2_Cu_3_O_7_ exhibits large peaks immediately below the Fermi energy level, which corroborates the theoretical perspectives discussed in the preceding section on feature interpretation.

Furthermore, the role of flat bands in superconductivity is frequently discussed alongside the concept of a Van Hove singularity in the DOS.^64,65^ A Van Hove singularity arises when the gradient of the band structure approaches zero, resulting in a pronounced peak in the DOS. The close proximity of such a singularity to the Fermi level can elevate *T*_c_ by intensifying the electronic interactions that facilitate superconductivity. Studies on various high-*T*_c_ cuprates have demonstrated that alterations in the band structure, induced by chemical doping or the application of pressure, can shift the relative positions of flat bands and Van Hove singularities with respect to the Fermi level, thereby affecting *T*_*c*_. This relationship highlights the importance of DOS profiles in understanding the electronic properties and behaviors that are essential to superconducting states.

#### 3.3.3 Case Study 3: Out-of-Distribution *T*_c_ Predictions for (Ca_1–*x*_La_*x*_)FeAs_2_ and (Ca_1–*x*_La_*x*_)Fe(As_1–*y*_Sb_*y*_)_2_

We expand upon the predictive capabilities of our ML model beyond benchmarking against state-of-the-art reports. Specifically, we undertook a case study, whereby we focused on predicting how the *T*_c_ value of (Ca_1–*x*_La_*x*_)FeAs_2_ varies with the lanthanum (La) concentration, denoted by *x*. For this analysis, the *T*_*c*_ values reported by Kudo et al.^66^ serve as the ground truth, while an additional 73 *T*_c_ records were sourced from Hosono et al.,^29^ expanding the training set. To ensure the integrity of these out-of-distribution predictions, we ensured that there was no overlap between the training and test sets. Here, out-of-distribution predictions refer to model predictions that are made on chemical compositions that are either underrepresented or entirely absent from the training data. This approach challenges our ML model to navigate unexplored material spaces by extrapolating from established chemical-property relationships learned during training. This endeavor not only tests the robustness of our model but also enhances our understanding of its predictive power and potential utility in practical applications.

**Figure 19:**
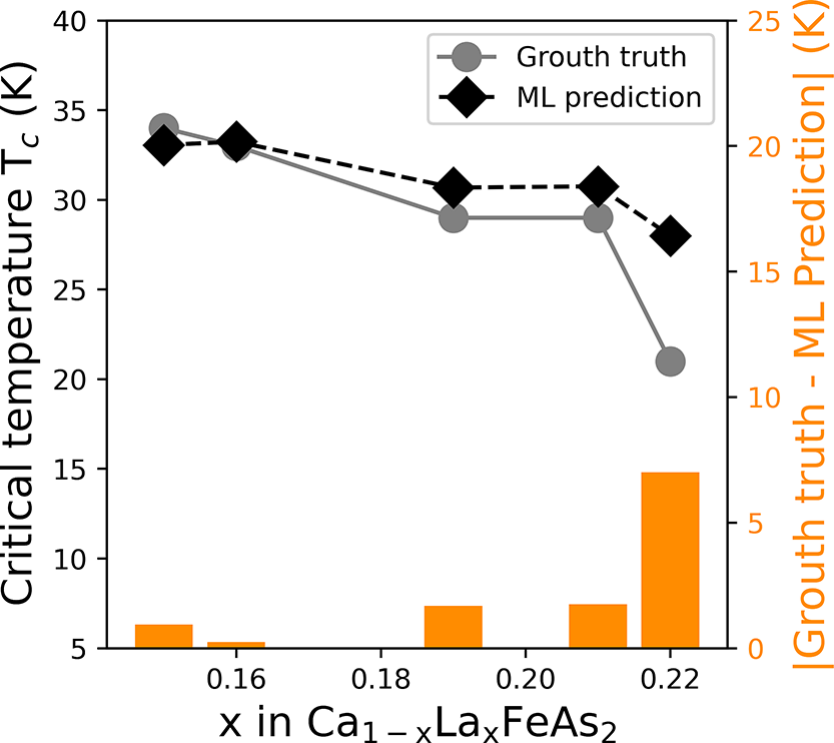
ML-based predictions of the superconducting transition temperature *T*_c_ of (Ca_1–*x*_La_*x*_)FeAs_2_ on the La content *x* against literature values.^66^ Orange bar chart illustrates the residuals, defined as the absolute discrepancies between the predicted values (black diamonds) and the grouth truth (gray circles) measurements.

Figure 19 presents the ML-based predictions of the superconducting transition temperature *T*_c_ for (Ca_1–*x*_La_*x*_)FeAs_2_ as a function of the lanthanum (La) content (*x*), juxtaposed with literature values. The predictions illustrate *T*_c_ as calculated based on the proportional composition of La. The figure includes an orange bar chart that depicts the residuals, which are defined as the absolute differences between the ML-predicted values (represented by black diamonds) and the ground-truth measurements (depicted by gray circles). This visualization highlights the levels of accuracy and discrepancies of the model’s predictions relative to established empirical data. The results indicate that the majority of the *T*_c_ predictions made by our ML model for the previously unseen chemical compositions in this family of iron-based superconductors correspond closely with values reported in the literature, effectively capturing the overall downward trend of *T*_c_ as the La content increases to 0.22. The 112 phase of the chemical family, (Ca_1–*x*_La_*x*_)FeAs_2_, is achievable at *x* ≥ 0.15. Notably, the *T*_c_ value is at its highest with 35 K when the composition, *x*, is at its lower boundary of *x* = 0.15; moreover, *T*_c_ decreases monotonically with increasing La content.

While not depicted in Figure 19, additional computations were performed for another case study that involved an antimony (Sb)-doped variant of this chemical family, whose generic chemical formula is (Ca_1–*x*_La_*x*_)Fe(As_1–*y*_Sb_*y*_)_2_, where we specifically focused on a compound with *y* = 0.01 and *x* = 0.15. Our ML model predicted a *T*_c_ value of 40 K for this compound, which closely aligns with the 43 K reported by Kudo et al.^66^ This observation reinforces the *T*_c_ trend and it is attributable to the introduction of Sb doping. In particular, simultaneous Sb doping facilitates a reduction in La content down to *x* = 0.12, yielding an increase in *T*_c_ to 47 K. Generally, such chemical substitutions not only modify the number of charge carriers but also induce a form of chemical pressure, influencing the electronic properties and superconducting behavior of the material.

The enhancement of *T*_c_ in the (Ca_1–*x*_La_*x*_)Fe(As_1–*y*_Sb_*y*_)_2_ family of chemicals can be attributed to two key factors. First, the reduction in La content influences *T*_c_ due to its role in modifying charge carrier density. Kudo et al. found that, despite the doping of La, the unit cell volume and lattice parameters of (Ca_1–*x*_La_*x*_)FeAs_2_ remain largely unchanged, which is attributed to the similar ionic radii of La^3+^ and Ca^2+^.^66^ This observation indicates that a reduction in La content directly depletes the number of charge carriers. Second, the incorporation of Sb introduces a distinct effect. Simultaneous Sb doping exerts a negative chemical pressure, leading to an increase in unit cell volume.^29,66^ This is due to the fact that the ionic radius of As^3–^ is smaller than that of Sb^3–^. The resultant increase in unit cell volume primarily affects the in-plane crystallographic lattice parameters *a* and *b*, while the *c* parameter remains relatively stable. This analysis underscores the complex interplay of features that influence the prediction of *T*_c_ in superconductors, demonstrating that *T*_c_ is highly contingent upon a multitude of factors.

#### 3.3.4 Case Study 4: Virtual Compositional Search of Hg–Pb–Ba–Ca–Cu–O Series of Compounds

In our fourth case study, we explored the potential utility of ML models to assist in the compositional search for a particular set of chemical compounds. Specifically, we examined whether ML models could be employed to modify the doping levels of certain chemical elements to predict or identify trends in material properties, such as critical temperature of superconductivity, *T*_c_. Herein, we focus on Hg–Pb–Ba–Ca–Cu–O series of compounds, starting from the compound with chemical composition Hg_0.66_Pb_0.34_Ba_2_Ca_1.98_Cu_2.9_O_8.4_, which holds the record for the highest *T*_c_ value within the Supercon database. In this chemical compound, the elements mercury (Hg) and lead (Pb) are typically considered to be dopants. While Hg is a part of the main structure in some high-temperature superconductors, its fractional composition (i.e., 0.66) in this specific chemical stoichiometry suggests a significant role in optimizing its crystal lattice structure or enhancing its superconducting properties, possibly through partial substitution or supplementation of other elements. Moreover, Pb, indicated by the stoichiometric fraction 0.34, is likely serving a doping function aimed at modifying material properties to improve material stability or modify electronic structures, with an aim to enhance *T*_c_.

**Figure 20:**
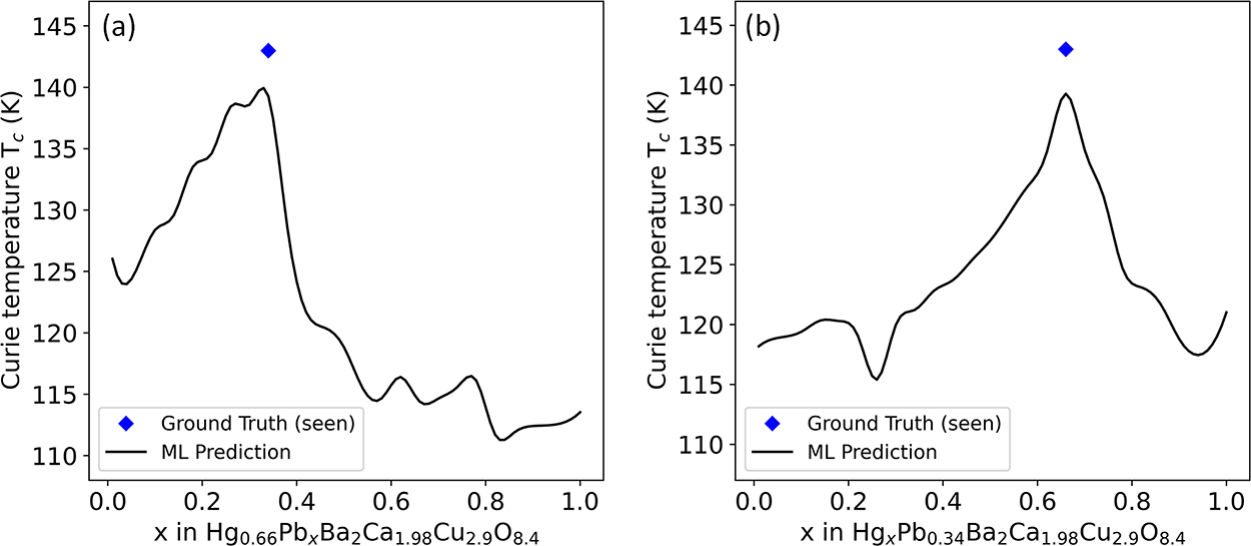
ML-based virtual compositional analysis of Hg–Pb–Ba–Ca–Cu–O series of compounds through independent and systematic variation of the concentrations of (a) Pb and (b) Hg to explore potential trends in the *T*_c_ profiles of virtual chemical compounds.

To investigate these hypotheses, we performed a virtual compositional search by independently adjusting the concentrations of Hg and Pb in a systematic manner, with an increment of 0.01 in the elemental content (*x*), to identify possible trends in *T*_c_ profiles. It is essential to underscore that this analysis presupposes the chemical validity and stability (i.e., synthesizability) of all variants, without consideration of any specific structural forms or alterations. Our method focused exclusively on modifying the chemical composition to establish a predictive profile of *T*_c_ for these virtual compounds. Consequently, the findings should be interpreted cautiously, serving more as an illustration of the potential utility of ML models rather than as definitive results. The outcomes of this investigation are depicted in Figure 20.

The marked sensitivity of *T*_c_ to variations in the Hg and Pb concentrations indicates that these elements are crucial to the superconducting properties of the material, supporting the fact that achieving optimal doping levels is essential for maximizing *T*_c_. As anticipated, in both cases, the ML model identified a single pronounced peak near the ground truth (starting compound), which was seen by the ML model during the training phase, with no other significant peaks observed. This potentially suggests the absence of other chemical variants with *T*_c_ values which are comparable to that of Hg_0.66_Pb_0.34_Ba_2_Ca_1.98_Cu_2.9_O_8.4_, at least within the compositional space in which we have searched herein. In Figure 20a, variations in the Pb content (*x*) resulted in a profile showing relatively greater fluctuations in the *T*_c_ profile, potentially indicating complex interactions or phase transitions within the virtual superconductor that impacts its superconductivity. Moreover, a notable decrease followed by smaller peaks in *T*_c_ as Pb concentration increases beyond the primary peak may suggest possible instability or variations in superconducting properties driven by chemical composition modifications. Conversely, Figure 20b presents a smoother decline in *T*_c_ after the peak, implying that variations in the Hg content might lead to a more gradual adjustment in superconducting properties compared to changes in the fractional content of Pb. This differential response underscores the intricate relationship between chemical composition and the superconducting characteristics of the material.

Despite the fact that this chemical compound search is based on a systematic virtual compositional survey, which relies on several scientifically unwarranted assumptions as previously noted, these results nevertheless illustrate the potential utility of ML models to perform compositional searches around an experimentally verified chemical material to predict superconducting properties from chemical compositions. This could be pivotal for designing new superconductors without extensive empirical testing. Insights from analysis such as these could guide the synthesis of new superconductors with tailored properties by adjusting the concentrations of dopants within a compound. Moreover, these figures illustrate the fundamental role of elemental composition in determining the superconducting properties of cuprate superconductors. They also demonstrate the utility of ML in forecasting material properties based exclusively on chemical compositions, potentially expediting the discovery and refinement of new superconducting materials.

#### 3.3.5 Potential Enhancements for Prediction Assessment and Modeling

In concluding this section, we note that an additional evaluation step could be implemented to further assess the predicted *T*_c_ values. High-throughput calculations or simulations could be incorporated as an extra evaluation step into our GBFS workflow. For instance, computational simulations of materials and their properties have streamlined the process of material characterization and behavior analysis, allowing for the exploration of vast chemical and property spaces, as well as predicting material behavior across various research domains.^67−70^ Such an approach would provide supplementary validation of the predictions made by the ML model, particularly given the limited availability of experimental measurements for *T*_c_. Currently, our modeling approach relies exclusively on experimental data, as they are considered to be the ground truth. Although incorporating a DFT-evaluation step could strengthen the robustness of the predictions, it is important to recognize that computational calculations such as those from DFT, though valuable, inherently contain errors due to their approximate nature. Additionally, such an inclusion would necessitate a greater computational resource. Nonetheless, we have suggested this additional evaluation step for future research, acknowledging the challenges posed by the limited availability of experimental measurements in superconductivity studies.

Building on this suggestion, it is pertinent to discuss Machine Learning Interatomic Potentials (MLIPs).^71,72^ These advanced computational models employ ML techniques to determine the potential energy surface of materials based on their atomic configurations. MLIPs effectively bridge the gap between the high fidelity of first-principles calculations, such as DFT, and the computational efficiency needed for large-scale simulations. This capability renders them especially beneficial for the investigation of materials where conventional methods would demand excessive computational resources. MLIPs excel at modeling intricate atomic interactions more effectively than traditional potential models, positioning them as excellent tools for probing new materials. These models are adaptable to various data sets, facilitating the exploration of diverse material properties without requiring constant recalculations from first-principles methods. The utilization of MLIPs has the potential to propel advancements in materials science, enhancing the efficiency and accuracy of simulations, especially in the discovery and characterization of materials with complex properties.

## 4 Conclusions

We have developed and implemented a machine learning-based workflow for feature selection and statistical analysis, with the aim of realizing ML models that accurately predict the critical temperature (*T*_c_) of superconductors. Our proposed methodology, named the GBFS workflow, employs a distributed gradient-boosting framework that synergistically combines exploratory data analyses, statistical evaluations, and multicollinearity reduction techniques. This integrated approach effectively identifies and selects a targeted subset of features within a high-dimensional feature space, focusing on those with high relevance to the target variable or class while minimizing feature redundancy. The selected features, derived exclusively from the chemical composition of materials, are then employed to train and fine-tune the hyperparameters of gradient-boosting trees using Bayesian optimization.

In a comprehensive analysis of approximately 16,400 chemical compounds, encompassing around 12,000 unique chemical compositions, our GBFS workflow facilitated the development of a classification model that is capable of discriminating chemical compositions that are likely to exhibit a *T*_c_ value greater than 10 K. This model achieved a weighted average F1-score of 0.912, an AUC-ROC of 0.986, and an average precision score of 0.919. Moreover, we further leveraged the GBFS workflow, to produce a regression model that predicts *T*_c_ values with an *R*^2^ of 0.945, an MAE of 3.54 K, and an RMSE of 6.57 K on a test set derived through random splitting.

We extended the applications of our ML models beyond benchmarking against state-of-the-art reports. This exploration included a detailed examination of out-of-sample *T*_c_ predictions, specifically those exceeding the liquid-nitrogen temperature. This included the evaluation of 50 randomly selected compounds for which an experimentally measured superconducting *T*_c_ value above 85 K has been reported in each case. Our ML model predicted *T*_c_ values that lie within 8% of the experimental measurements, for 44 of these 50 compounds. This demonstrate the efficacy of our modeling approach. Additionally, we performed out-of-distribution *T*_c_ predictions of (Ca_1–*x*_La_*x*_)FeAs_2_ as a function of lanthanum (La) content, thereby showcasing the model’s ability to extrapolate chemical-property relationships learned from the materials database. These results further validate the efficacy of our modeling approach and emphasize the importance of meticulous feature analysis and selection, conducted via a systematic manner, in enhancing model performance. Our modeling approach contrasts sharply with strategies that rely predominantly on algorithmic complexity.

## Data Availability

We have made available the data and the code for the feature selection, statistical analyses, multicollinearity reduction, recursive-feature elimination and Bayesian optimization at https://github.com/Songyosk/SuperconductivityML.

## Supporting Information Available

Pseudo-code for Bayesian optimization and regression analysis using the ‘all-Tc’ model (PDF)
Crystallographic information of test-set errors (XLSX)
