# Supplementary material for: Machine-Learning Predictions of Critical Temperatures from Chemical Compositions of Superconductors
Source: J Chem Inf Model. 2024 Sep 17;64(19):7349–75. doi: 10.1021/acs.jcim.4c01137 (PMC11481088; doi:10.1021/acs.jcim.4c01137)
Supplement: Supplementary file 1 — ci4c01137_si_001.pdf [file ci4c01137_si_001.pdf]

# Supporting Information

# Machine-Learning Predictions of Critical Temperatures from Chemical Compositions of Superconductors

Son Gyo Jung[1,2,3], Guwon Jung[1,3,4], Jacqueline M. Cole[1,2,3,*]

[1]*Cavendish Laboratory, Department of Physics, University of Cambridge, J. J. Thomson Avenue, Cambridge, CB3 0HE, U.K.*

[2]*ISIS Neutron and Muon Source, STFC Rutherford Appleton Laboratory, Harwell Science and Innovation Campus, Didcot, Oxfordshire, OX11 0QX, U.K.*

[3]*Research Complex at Harwell, Rutherford Appleton Laboratory, Harwell Science and Innovation Campus, Didcot, Oxfordshire, OX11 0FA, U.K.*

[4]*Scientific Computing Department, STFC Rutherford Appleton Laboratory, Harwell Science and Innovation Campus, Didcot, Oxfordshire, OX11 0QX, U.K.*

[*]jmc61@cam.ac.uk

## SI.1 Pseudo-code for Bayesian optimization

---

**Algorithm 1:** Bayesian optimization with Gaussian process prior

---

**input:** objective function $f$, hyper-parameter space $\boldsymbol{\theta}$, acquisition functions $\alpha$,

initialization points $T_{init}$, maximum number of evaluation $T$

$y_{best} \leftarrow 0$ ;

**for** $t = 1$ **to** $T_{init}$ **do**

  select $\boldsymbol{\theta}_t$ via randomly sampling;

  compute exact objective function $y_t \leftarrow f(\boldsymbol{\theta}_t)$;

  **if** $y_t > y_{best}$ **then**

    $\boldsymbol{\theta}_{best} \leftarrow \boldsymbol{\theta}_t$;

    $y_{best} \leftarrow y_t$;

  **end**

**end**

**for** $t = T_{init} + 1$ **to** $T$ **do**

  build probabilistic model for $f$ conditioned on previous observations $D_{1:t-1}$;

  compute all possible true functions using Gaussian process regression;

  optimize acquisition functions $\alpha$ independently based on the posterior distribution

  and propose a candidate point for each acquisition scheme

  $\boldsymbol{\theta}_{t,s} \leftarrow argmax_{\boldsymbol{\theta}} \ \alpha_s(\boldsymbol{\theta}|D_{1:t-1})$ for $s = \{PI, EI, UCB\}$;

  choose next evaluation point $\boldsymbol{\theta}_t \leftarrow argmax_{\boldsymbol{\theta}} \ softmax(\mu(\boldsymbol{\theta}_{t,s}))$;

  compute exact objective function $y_t \leftarrow f(\boldsymbol{\theta}_t)$;

  **if** $y_t > y_{best}$ **then**

    $\boldsymbol{\theta}_{best} \leftarrow \boldsymbol{\theta}_t$

    $y_{best} \leftarrow y_t$

  **end**

**end**

**return** $\boldsymbol{\theta}_{best}$

---

Three acquisition functions, denoted by $\alpha$, each correspond to one of the following acquisition schemes: (i) Probability of Improvement (PI), (ii) Expected Improvement (EI), and (iii) Upper-Confidence-Bounds (UCB).

# SI. 2 - Regression Analysis Using the 'all-$T_c$' Model

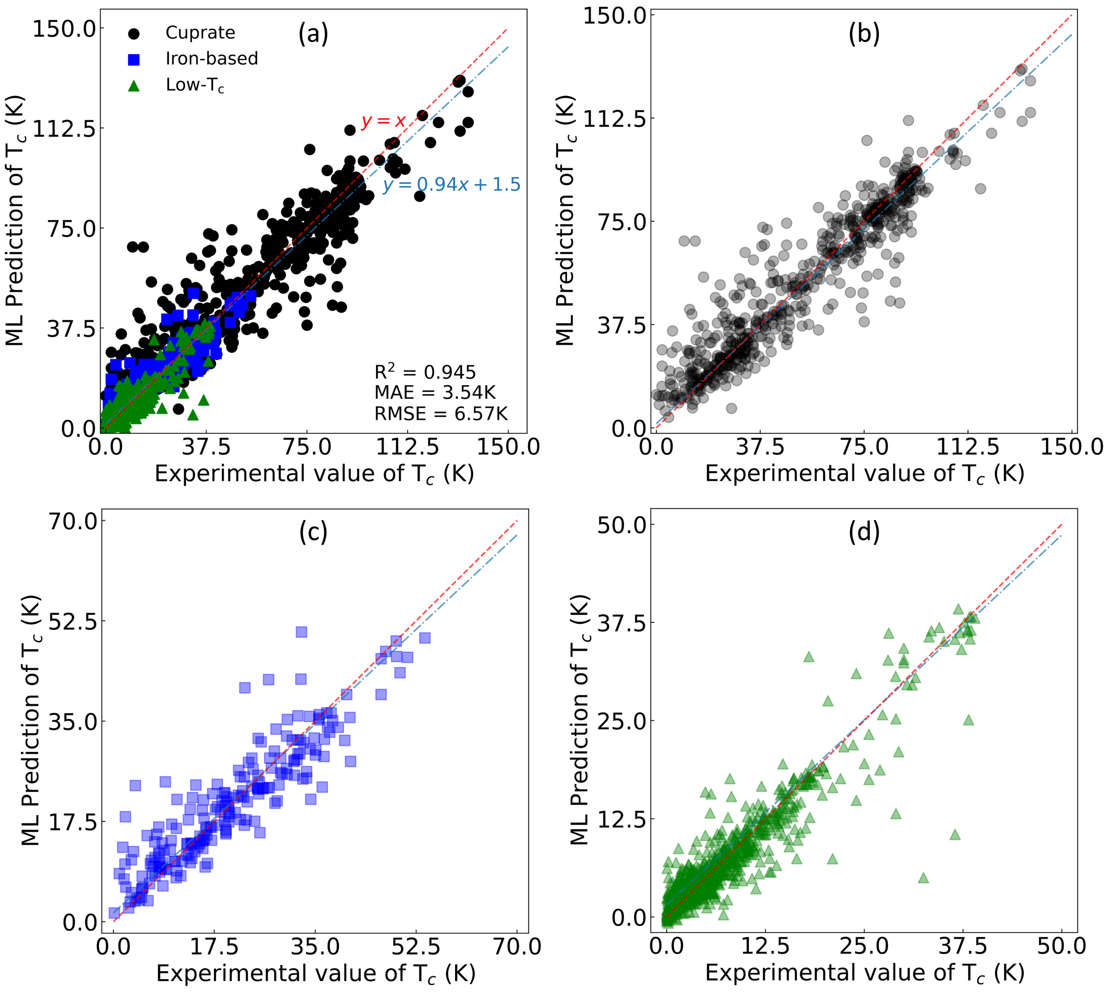


Figure S1.1: ML-based predictions of critical temperature ($T_c$) plotted against experimental values (ground truth) for the 'all-$T_c$' model. Panel (a) displays the combined results for all superconductors, while panels (b), (c), and (d) specifically isolate cuprate, iron-based, and low-$T_c$ superconductors, respectively. These plots represent the results from the out-of-sample test set.
